# Exploring the Inhibitory Potential of Six Porphyrin Compounds Against α-Amylase and α-Glucosidase Linked to Diabetes

**DOI:** 10.3390/biom15091338

**Published:** 2025-09-18

**Authors:** Shuo Zhang, Zi Liu, Qiurui Ma, Yangyuxin Liu, Shuren Yin, Zhihan Zhou, Jie Zhou, Helong Bai, Tianjiao Li

**Affiliations:** College of Chemistry, Changchun Normal University, Changchun 130032, China; 210414020338@stu.ccsfu.edu.cn (S.Z.); 2309010213@stu.ccsfu.edu.cn (Z.L.); 190419040119@stu.ccsfu.edu.cn (Q.M.); 2309010211@stu.ccsfu.edu.cn (Y.L.); 220414020231@stu.ccsfu.edu.cn (S.Y.); 2311030133@stu.ccsfu.edu.cn (Z.Z.); zhoujie001119@163.com (J.Z.)

**Keywords:** porphyrin compound, inhibition, α-amylase, α-glucosidase

## Abstract

Diabetes mellitus is a characteristic metabolic disorder with diverse complications. α-Amylase and α-glucosidase, as key digestive enzymes regulating blood glucose, are important targets for diabetes prevention and management through their inhibition. This study investigated the inhibitory effects of six porphyrin compounds (TAPP, TCPP, THPP, Cu–TCPP, Fe–TCPP, Ni–TCPP) on two enzymes through in vitro inhibition assays, spectroscopic experiments, and molecular docking techniques. All six compounds effectively inhibited the activities of both enzymes. For α-amylase, the inhibitory potency (IC_50_ = 13.03–245.04 μg/mL) followed the order TAPP > THPP > TCPP > Fe–TCPP > Ni–TCPP > Cu–TCPP. All six compounds exhibited more potent inhibitory activity against α-glucosidase (IC_50_ = 0.24–25.43 μg/mL), with potency in the order of THPP > Ni–TCPP > Fe–TCPP > TCPP > Cu–TCPP > TAPP. Fluorescence quenching experiments revealed that all compounds statically quenched the intrinsic fluorescence of both enzymes (with Fe–TCPP exhibiting static-dominant mixed quenching against α-amylase), indicating complex formation. These interactions significantly altered the enzymes’ conformations, the microenvironments of Tyr/Trp residues, and secondary structure content, consequently reducing their catalytic activity. By examining the inhibitory impact of porphyrin compounds on α-amylase and α-glucosidase, this research establishes a vital experimental and theoretical basis for diabetes therapeutics.

## 1. Introduction

Diabetes, as a lifelong, progressive chronic metabolic disorder [1], now ranks among the fastest-growing non-communicable diseases [2,3]. Its pathological characteristics manifest as persistent hyperglycemia resulting from impaired carbohydrate metabolism caused by insulin dysfunction [4,5]. Clinically, it presents with symptoms like polydipsia, polyuria, polyphagia, and weight loss. This chronic metabolic disturbance can induce [6] structural damage and functional impairment in multiple organ systems (including the visual, cardiovascular, urinary, and nervous systems), ultimately increasing patients’ mortality risk due to complications. The extent of organ damage caused by hyperglycemia is closely associated with both the duration of the disease and the degree of glycemic control [7]. Diabetes has evolved into a major global public health challenge, imposing a heavy economic burden on patients’ families and societal healthcare systems.

Diabetes encompasses multiple forms, with type 1 diabetes (T1DM) and type 2 diabetes (T2DM) being the most prevalent [8]. T1DM is caused by damage to β cells in the pancreatic islets, leading to insulin deficiency and high blood sugar [9], necessitating lifelong dependence on exogenous insulin therapy for patients. In contrast, the pathogenesis of T2DM arises from a disruption in the dynamic equilibrium between glucose metabolism and insulin secretion. This is specifically manifested as a progressive increase in blood glucose levels caused by insulin resistance and impaired β-cell function [10], collectively resulting in relative insulin insufficiency. T2DM accounts for over 90% of the global diabetic population [11,12] and has attracted significant attention due to its potential preventability. Maintaining blood glucose levels within the normal physiological range constitutes the primary therapeutic approach for T2DM [13]. A key factor triggering hyperglycemia is the hydrolytic breakdown of carbohydrates within the body [14]. Starch, a significant source of energy among carbohydrates, has its digestion rate and extent directly determining the magnitude of blood glucose elevation [15]. Consequently, delaying or inhibiting the starch digestion process can reduce the rate of glucose absorption in the small intestine, thereby mitigating the postprandial rise in blood glucose.

α-Amylase (α-AMY) and α-glucosidase (α-GLU) are key digestive enzymes regulating blood sugar after meals. In the digestive process of the human body, salivary and pancreatic α-amylase collectively participate in the hydrolysis of starch from the oral cavity to the small intestine [16]. α-Glucosidase is widely present in organisms that utilize carbohydrates as an energy source [17]. It functions as an exo-glycosidase, acting on the final step of the digestive process [18]. These enzymes break down complex carbohydrates into simple monosaccharides through enzymatic hydrolysis [19], which are subsequently absorbed into the bloodstream. By catalyzing the hydrolysis of dietary carbohydrates into monosaccharides in the intestine, these enzymes trigger postprandial blood glucose elevation. Inhibiting α-AMY and α-GLU activity reduces monosaccharide production, thereby decelerating glucose absorption and transport into the bloodstream [20]. Consequently, this approach not only effectively controls postprandial blood glucose levels but also holds significant importance for regulating blood glucose concentrations [21]. Additionally, α-glucosidase inhibitors can enhance insulin sensitivity [22]. Therefore, developing drugs capable of inhibiting the activity of α-AMY and α-GLU plays a positive role in the prevention and management of diabetes [23]. However, studies have found that while inhibiting α-amylase reduces glucose release, complete inhibition of this enzyme may cause intestinal disturbances. This occurs because the gut microbiota ferments the retained undigested starch, producing gases [24]. Thus, inhibiting α-AMY can reduce the rate at which starch is broken down into glucose, thereby suppressing rapid blood glucose elevation. Commercially available synthetic inhibitors used in the clinical treatment of diabetes include acarbose, miglitol, and voglibose. However, long-term use of acarbose is often accompanied by similar adverse gastrointestinal effects [25]. Therefore, in the development of novel inhibitors with reduced side effects and high cost-effectiveness, priority should be given to strategies combining α-amylase inhibition with α-glucosidase inhibition [26].

**Figure 1:**
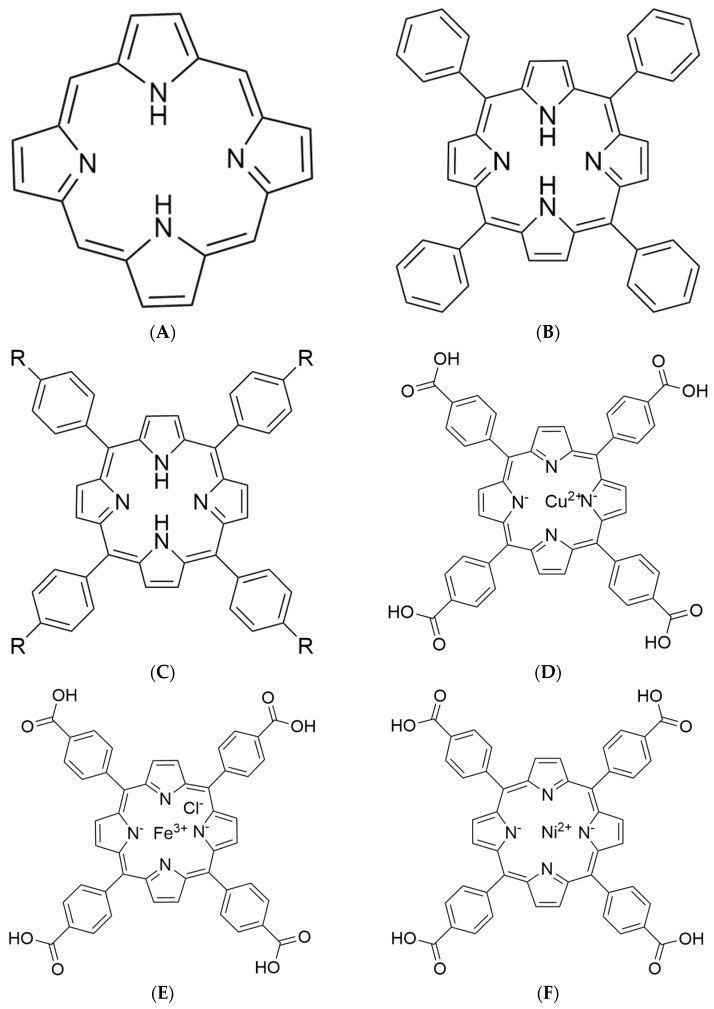
Structural formula of (**A**) porphyrin; (**B**) TPP; (**C**) TAPP: R = NH2; THPP: R = OH; TCPP: R = COOH; (**D**) Cu–TCPP. (**E**) Fe–TCPP; (**F**) Ni–TCPP.

Porphyrins are macrocyclic compounds containing four pyrrole molecules (Figure 1A). Its parent body is porphyrin; when the pyrrole protons in it are replaced by metal ions, it is metal porphyrin. Porphyrin compounds play an important role in life activities. Chlorophyll, heme, etc. are metal porphyrin compounds, which play a critical role in oxygen transfer, storage, activation, and photosynthesis. TPP is an aromatic macrocyclic compound formed by substituting the four meso-hydrogen atoms of the porphyrin core with phenyl groups. The aromatic compound TPP (Figure 1B) is formed by substituting four hydrogen atoms on the porphyrin core with phenyl groups. Subsequent functionalization of TPP’s phenyl rings with hydroxyl (-OH), amino (-NH_2_), or carboxyl (-COOH) groups yields derivatives with improved physicochemical properties, THPP, TAPP, and TCPP, respectively (Figure 1C).

In recent years, the use of metal complexes for the treatment of diabetes is a new treatment strategy [27]. Transition metal complexes have attracted attention for their insulin-mimicking effects and their inhibitory activity on α-AMY and α-GLU [28]. As an essential trace element, copper also has an important impact on insulin signaling pathways by regulating redox balance, enzyme activity, and signaling pathways in organisms. Excessive copper concentrations may trigger oxidative stress and inhibit phosphorylation of insulin receptors, which in turn leads to insulin-mediated glucose metabolism disorders, ultimately leading to insulin resistance and type 2 diabetes [29]. Iron [30], as a key component of hemoglobin and myoglobin, is not only involved in the transport and storage of oxygen but also provides energy for muscle activity, helping to sustain the basic function of immune cells and the normal redox reaction in the body. Nickel plays an important role in stabilizing DNA structure, regulating enzyme activity, and promoting red blood cell production while activating insulin coenzymes, thereby helping to lower blood sugar. Therefore, based on the unique structure of TCPP, incorporating copper (II), iron (III) and nickel (II) yields three metalloporphyrin compounds: copper (II) meso-tetra (4-carboxyphenyl)porphyrin (Cu-TCPP, Figure 1D), iron (III) meso-tetra (4-carboxyphenyl) porphyrin chloride (Fe-TCPP, Figure 1E) and nickel (II) meso-tetra (4-carboxyphenyl) porphine (Ni-TCPP, Figure 1F). Sankar et al. [31] studied the enzymatic inhibitory activity of the ligand N-[(3-phenoxyphenyl)methylene]-L-valine (HL) and its Ni and Cu complexes and found that [Ni(L)(H2O)_2_]·3/4H_2_O and [Cu(L)(H_2_O)_4_]·2H_2_O had stronger inhibitory activity on α-AMY and α-GLU than ligand HL. The iron complex of fisetin/quercetin [32] exhibits stronger α-glucosidase inhibition than its individual components, and all four compounds inhibit enzyme activity through mixed competition.

Therefore, this study selected three porphyrin compounds with different functional groups (TAPP, THPP, TCPP) and three metalloporphyrins incorporating different metal ions as central atoms (Cu–TCPP, Fe–TCPP, Ni–TCPP). A preliminary investigation was conducted on the interaction mechanisms between these six compounds and the enzymes α-amylase and α-glucosidase.

## 2. Materials and Methods

### 2.1. Materials

TCPP, α-amylase, α-glucosidase, pNPG, and acarbose were purchased from Shanghai Yuanye Biotechnology Co., Ltd. (Shanghai, China). Porphyrins and Gal-G2-α-CNP were purchased from Shanghai Maclean’s Biochemical Co., Ltd. (Shanghai, China).

### 2.2. Inhibitory Activity Assay

#### 2.2.1. α-Amylase Inhibitory Activity Assay

The inhibitory activity detection process was slightly modified, with reference [33] as the base method from the literature. Using acarbose as the positive control, the experimental groups were supplemented with varying concentrations of porphyrin compounds and the control group received 30 μL of phosphate buffer solution (pH 6.8) containing 0.05 mol/L NaCl. After thorough mixing, the samples were pre-incubated at 37 °C for 10 min. Subsequently, 100 μL of 5 mmol/L Gal-G2-α-CNP substrate was added to each sample. Following a 3 min reaction period, the absorbance of the solutions at 405 nm was measured using a microplate reader. The inhibition rate (%) was calculated based on the absorbance readings according to Formula (1).

(1) $$\mathrm{inhibition}\;\mathrm{rate}\%=({1-(A}_{1}{-A}_{2}{)/(A}_{3}{-A}_{4}))\times 100$$

In the formula, A_1_ represents the sample group; A_2_ represents the sample control group (where the enzyme was replaced with phosphate buffer); A_3_ represents the blank control group; and A_4_ represents the reagent blank control group (containing blank sample solution and phosphate buffer).

#### 2.2.2. α-Glucosidase Inhibitory Activity Assay

The assay for α-glucosidase inhibitory activity was slightly modified according to the method in the reference [34]. The experimental groups were supplemented with different concentrations of porphyrin compounds, followed by the addition of 100 μL of 5 mmol/L pNPG. After thorough mixing, the samples were pre-incubated at 37 °C for 10 min. Subsequently, the reaction was terminated by adding 0.1 mol/L of sodium carbonate (Na_2_CO_3_) solution. Following a 3 min reaction period, the absorbance of the solutions at 405 nm was measured using a microplate reader. The inhibition rate (%) was subsequently calculated using Formula (1).

#### 2.2.3. Calculation of IC_50_

Using the enzyme inhibition rate as the *y*-axis and the inhibitor concentration as the *x*-axis, the IC_50_ value was calculated through linear regression fitting with GraphPad Prism software (5.0).

### 2.3. Mode of α-AMY and α-GLU Inhibition

In the experiment, the concentration of the fixed substrate Gal-G2-α-CNP/pNPG solution was 5 mmol/L, and the concentrations of the two enzymes (100, 200, 300, 400 U/L) were used to determine the effect of different concentrations of porphyrins on enzyme activity. The concentration of the enzyme was 200 U/L, and the concentration of the substrate Gal-G2-α-CNP/pNPG solution (2.5, 5, 7.5, 10 mmol/L) was used to determine the effect of different concentrations of porphyrins on the enzyme-catalyzed substrate activity.

Upon identifying the type of inhibition, the kinetic equations vary for different inhibition types. For mixed-type inhibition, the kinetic equation is given by Formula (2); for non-competitive inhibition, it is described by Formula (3).

(2) $$\frac{1}{v}=\frac{K_{m}}{V_{\max}}\left(1+\frac{\left[I\right]}{K_{i}}\right)\frac{1}{\left[S\right]}+\frac{1}{V_{\max}}\left(1+\frac{\left[I\right]}{K_{is}}\right)$$

(3) $$\frac{1}{v}=\frac{K_{m}}{V_{\max}}\left(1+\frac{\left[I\right]}{K_{i}}\right)\frac{1}{\left[S\right]}+\frac{1}{V_{\max}}\left(1+\frac{\left[I\right]}{K_{i}}\right)$$

The inhibition constant for the free enzyme (*K_i_*) and the inhibition constant for the enzyme–substrate complex (*K_is_*) were calculated using Formulas (4) and (5), respectively.

(4) $$S\mathrm{lope}=\frac{K_{m}}{V_{\max}}+\frac{K_{m}[I]}{V_{\max}K_{i}}$$

(5) $$Y-\mathrm{int}ercept=\frac{1}{V_{\max}}+\frac{\left[I\right]}{V_{\max}K_{is}}$$

In the formula, *v*, *V*_max_, *K_m_*, *K_i_*, and *K_is_* represent the reaction rate, maximum reaction rate, Michaelis constant, and free enzyme inhibition constant, respectively, and the binding enzyme inhibition constant. [*S*] denotes the substrate concentration. [*I*] denotes the inhibitor concentration.

### 2.4. In Vitro Stability Studies

Samples of a certain mass concentration were subjected to a water bath at different temperatures (30, 40, 50, 60, 70, 80, 90, 100 °C) for 20 min and treated under different pH conditions (2.0, 3.0, 4.0, 5.0, 6.0, 7.0, 8.0, 9.0, and 10.0) for 20 min to determine the effect of temperature and pH on the stability of the two enzymes.

### 2.5. Fluorescence Quenching Method

In the α-amylase experiment, a series of porphyrins and amylase (200 U/L) were mixed in a volume ratio of 1:1. In the α-glucosidase experiment, the glucosidase concentration was 400 U/L, mixed in a volume ratio of 1:1; the samples were maintained at three different temperatures (298, 304, and 310 K) for 30 min, with excitation at 280 nm and emission scanning from 300 to 500 nm.

The Stern–Volmer equation [35] was adopted to determine the quenching mechanisms between six porphyrin compounds and both enzymes in the experimental group, with Formula (6):

(6) $$\frac{F_{0}}{F}=1+K_{sv}[Q]=1+K_{q}\tau_{0}[Q]$$

The Stern–Volmer equation was adapted based on experimental data to more accurately characterize the quenching behavior [36], with Formula (7):

(7) $$\frac{F_{0}}{F}=e^{K_{sv}[Q]}=e^{K_{q}\tau_{0}[Q]}$$

In the formula, the fluorescence quenching ratio was calculated as *F*_0_*/F*, where *F*_0_ corresponds to the emission intensity of pure α-amylase solution (control) and *F* denotes the intensity measured after the incremental addition of porphyrin ligands. *K_SV_* and *K_q_* denote the Stern–Volmer quenching constant and the bimolecular quenching rate constant, respectively, τ_0_ (10^−8^ s) is the average fluorescence lifetime of the fluorophore in the absence of quencher, and [*Q*] indicates the concentration of porphyrin compounds.

Due to the presence of an internal filtration effect, a decrease in the fluorescence intensity of the system may occur. Therefore, all fluorescence data were corrected using Formula (8) to eliminate the internal filtration effect [37].

(8) $$F_{cor}=F_{obs}e^{\frac{\left(A_{ex}+A_{em}\right)}{2}}$$

In the formula, *F_cor_* and F*_obs_* represent the corrected and observed fluorescence intensities, respectively, while *A_ex_* and *A_em_* denote the absorption values of the system at the excitation and emission wavelengths, respectively.

The evaluation of the interaction forces between porphyrin compounds and the two enzymes can be achieved by calculating thermodynamic parameters. When both ΔH^0^ and ΔS^0^ are positive, the predominant interaction force is hydrophobic. When both ΔH^0^ and ΔS^0^ are negative, the main forces are hydrogen bonding and van der Waals forces. When ΔH^0^ < 0 and ΔS^0^ > 0, the primary interaction is electrostatic attraction [38].

The thermodynamic parameters were calculated using Formulas (9) and (10), and then the Van’t Hoff plot for the interactions between the porphyrin compounds and the two enzymes was generated by plotting ln*K*_a_ on the *Y*-axis versus 1/*T* on the *X*-axis.

(9) $$\ln K_{a}=-\frac{{\Delta H}^{0}}{RT}+\frac{{\Delta S}^{0}}{R}$$

(10) $${\Delta G}^{0}={\Delta H}^{0}-T{\Delta S}^{0}=-RT\ln K_{a}$$

### 2.6. Synchronous Fluorescence Spectroscopy

Synchronous fluorescence spectra (200–400 nm, Δλ = 15/60 nm) were acquired at 298 K to probe alterations in the microenvironments of tryptophan (Trp) and tyrosine (Tyr) residues induced by inhibitor binding, with spectral shifts interpreted in terms of polarity and hydrophobicity changes [39,40].

The synchronous fluorescence quenching ratio or RSFQ was calculated using Formula (11) to compare the contributions of Tyr and Trp residues to the fluorescence quenching of six porphyrin compounds with amylase and glucosidase.

(11) $$RSFQ=1-\frac{F}{F_{0}}$$

### 2.7. Three-Dimensional Fluorescence Spectroscopy

At 298 K, with excitation wavelengths ranging from 200 to 600 nm (2 nm increments) and emission wavelengths from 200 to 600 nm, the influence of small molecules on enzyme conformation was assessed by examining peaks in 3D fluorescence spectra [41].

### 2.8. Fourier Transform Infrared Spectroscopy

A molar ratio of 1:2 (α-amylase/α-glucosidase)–porphyrin solution was placed in a cryocentrifugal concentrator and the dried solid sample was mixed with dry KBr and measured using a Nicolet IS-50 FTIR Spectrometer (ThermoFisher, Waltham, MA, USA) with a scanning range of 4000–400 cm^−1^ and a resolution of 4 cm^−1^. The overlapping peaks in the amide I band (1700–1600 cm^−1^) were further analyzed into multiple feature sub-peaks, and quantitative analysis of the relative content of various secondary structures in proteins using curve fitting methods was performed [42].

### 2.9. Molecular Docking

The crystal structures of α-amylase (PDB ID: 1OSE) and α-glucosidase (PDB ID: 5ZCB) from the RCSB Protein Data Bank (RCSB PDB) were downloaded. Using the Protein Preparation Workflow module in Schrödinger software (2019), hydrogens were added, charges were assigned, and the protonation states and hydrogen bonding networks of the proteins and ligands were optimized. The optimized lowest-energy conformation of the ligand was used as the input structure for docking. Molecular docking was performed using the Glide XP (extra precision) mode. The potential binding modes of porphyrin-based compounds with α-amylase and α-glucosidase were analyzed.

### 2.10. Molecular Dynamics Simulation

Using the Amber ff19SB force field parameters in Amber 24, the system was placed in a TIP3P water model, and Na^+^ ions were added to neutralize the total charge of the system. After energy minimization, density equilibration was maintained for 500 ps at a constant temperature of 300 K and a pressure of 1.0 atm. Finally, a final production simulation of 100 ns was performed for each system at 300 K.

### 2.11. Cytotoxicity Assay

The cytotoxicity of six porphyrin compounds against human hepatocellular carcinoma HepG2 cells was evaluated using the MTT assay. HepG2 cells were seeded into 96-well plates at a density of 5000 cells per well and cultured for 24 h at 37 °C in a 5% CO*_2_* incubator. Then, 100 µL of sample solutions at various concentrations (50, 100, and 200 µg/mL) was added to the experimental wells, with blank control wells and solvent control wells also included. The cells were further incubated for 24 h. After incubation, 10 µL of MTT solution (5 mg/mL) was added to each well, and the plates were incubated for another 4 h. Subsequently, 100 µL of Formazan solubilization solution was added to each well, followed by gentle mixing. The plates were returned to the incubator and maintained until complete dissolution of Formazan crystals was observed under a microscope. The absorbance at 570 nm was measured using a microplate reader (Meigu Molecular Instrument Company, Shanghai, China), and the survival rate of HepG2 cells was calculated.

(12) $$\mathrm{Cell}\;\mathrm{viability}=\frac{A_{s}{-A}_{b}}{A_{c}{-A}_{b}}\times 100\%$$

In the formula, *A*_s_ represents the absorbance of the sample well, A_c_ denotes the absorbance of the blank control well (without the drug), and A_b_ refers to the absorbance of the solvent control well (devoid of both cells and the drug).

### 2.12. Pharmacokinetic Analysis

Using the admetSAR 2.0 online platform (http://lmmd.ecust.edu.cn/admetsar2, accessed on 4 August 2025) for pharmacokinetic prediction, the absorption, distribution, metabolism, excretion, and toxicity of compounds in the body were analyzed. The human intestinal absorption rate (HIA), blood–brain barrier permeability (BBB), and acute oral toxicity were employed to evaluate the medicinal and edible safety as well as the digestive and metabolic effects of the compounds.

### 2.13. Statistical Analysis

Three parallel experiments were performed for each group of experiments, and the experimental results were plotted using Origin 2018 and statistically analyzed with SPSS 19.0 software. All experimental data are expressed as x ± s.

## 3. Results

### 3.1. Inhibition of α-AMY and α-GLU by Six Porphyrin Compounds

**Figure 2:**
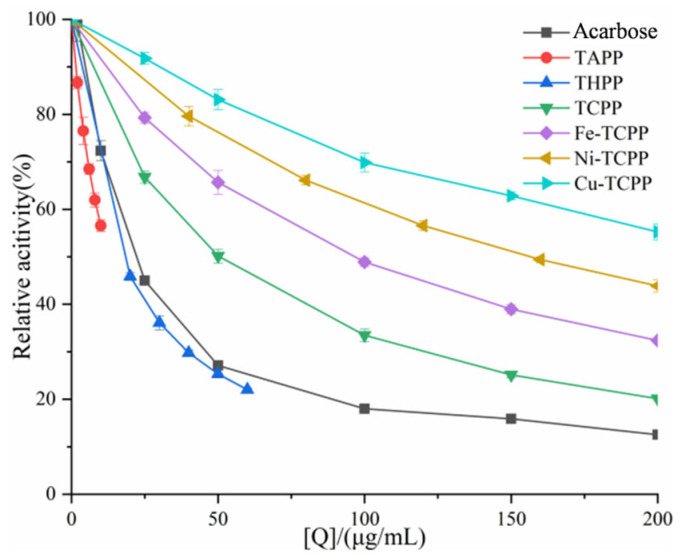
Inhibitory effect of porphyrin compounds on α-AMY activity. Q is the sample concentration.

**Figure 3:**
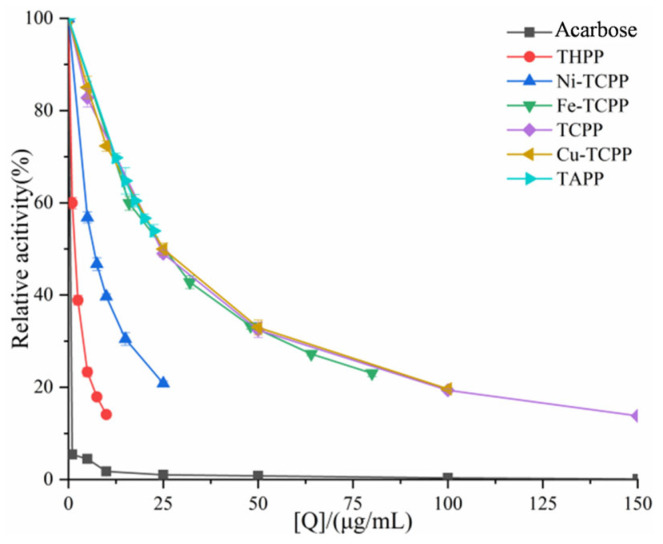
Inhibitory effect of porphyrin compounds on α-GLU activity. Q is the sample concentration.

The inhibitory ability of six porphyrin compounds on α-AMY and α-GLU was studied by establishing an in vitro viability assay system. As shown in Figure 2 and Figure 3, the inhibitory activity of the compounds against α-AMY increased with higher sample concentrations, while a decreasing trend was observed for α-GLU. These porphyrins and the positive control acarbose had the same concentrations of inhibition of α-amylase as TAPP (IC_50_ = 13.03 ± 0.62 μg/mL) > THPP (IC_50_ = 16.95 ± 0.56 μg/mL) > acarbose (IC_50_ = 23.20 ± 0.64 μg/mL) > TCPP (IC_50_ = 50.32 ± 0.82 μg/mL) > Fe–TCPP (IC_50_ = 95.75 ± 0.62 μg/mL) > Ni–TCPP (IC_50_ = 156.26 ± 0.48 μg/mL) > Cu–TCPP (IC_50_ = 245.04 ± 0.26 μg/mL). The inhibition rate of α-GLU was as follows: acarbose (IC_50_ = 0.24 ± 0.02 μg/mL) > THPP (IC_50_ = 1.53 ± 0.04 μg/mL) > Ni–TCPP (IC_50_ = 6.58 ± 0.21 μg/mL) > Fe–TCPP (IC_50_ = 23.93 ± 0.53 μg/mL) > TCPP (IC_50_ = 24.00 ± 0.56 μg/mL) > Cu–TCPP (IC_50_ = 25.33 ± 0.55 μg/mL) > TAPP (IC_50_ = 25.43 ± 0.54 μg/mL). The above results indicate that compared to acarbose, TAPP and THPP are superior α-amylase inhibitors, but they exhibit weaker inhibitory effects on α-glucosidase activity.

It should be noted that the IC_50_ values calculated here do not account for the type of inhibition. For complex inhibition mechanisms, IC_50_ may not directly reflect the true inhibitory potency and must therefore be interpreted in conjunction with enzymatic kinetic parameters. Thus, these initial IC_50_ values serve as a preliminary guide to inhibitory potential. Further validation is required in subsequent kinetic analyses to determine whether the order of inhibition is consistent with the IC_50_ values.

### 3.2. Inhibition Modes of Six Porphyrin Compounds Against Two Enzymes

**Figure 4:**
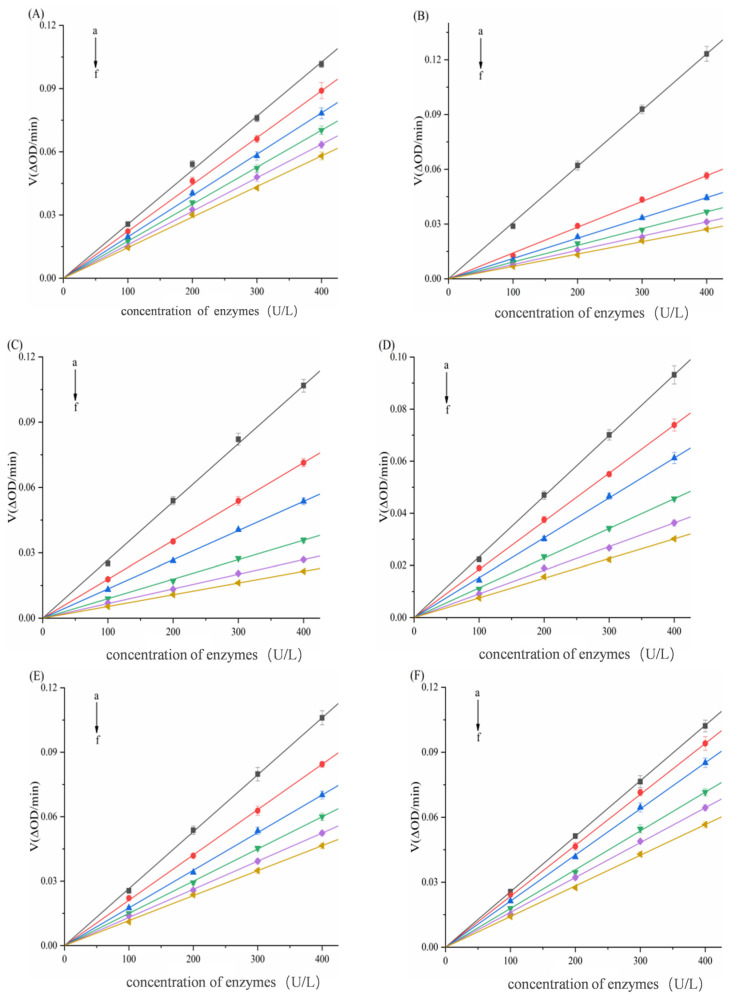
Analysis of reversible inhibition of α-amylase by six porphyrins. (**A**) TAPP, (**B**) THPP, (**C**) TCPP, (**D**) Fe–TCPP, (**E**) Ni–TCPP, (**F**) reversibility of Cu–TCPP binding to α-amylase a→f is judged to be (**A**) c (TAPP) = 0, 2, 4, 6, 8, 10 μg/mL; (**B**) c (THPP) = 0, 20, 30, 40, 50, 60 μg/mL; (**C**) c (TCPP) = 0, 25, 50, 100, 150, 200 μg/mL; (**D)** c (Fe–TCPP) = 0, 25, 50, 100, 150, 200 μg/mL; (**E**) c (Ni–TCPP) = 0, 40, 80, 120, 160, 200 μg/mL; (**F**) c (Cu–TCPP) = 0, 25, 50, 100, 150, 200 μg/mL.

**Figure 5:**
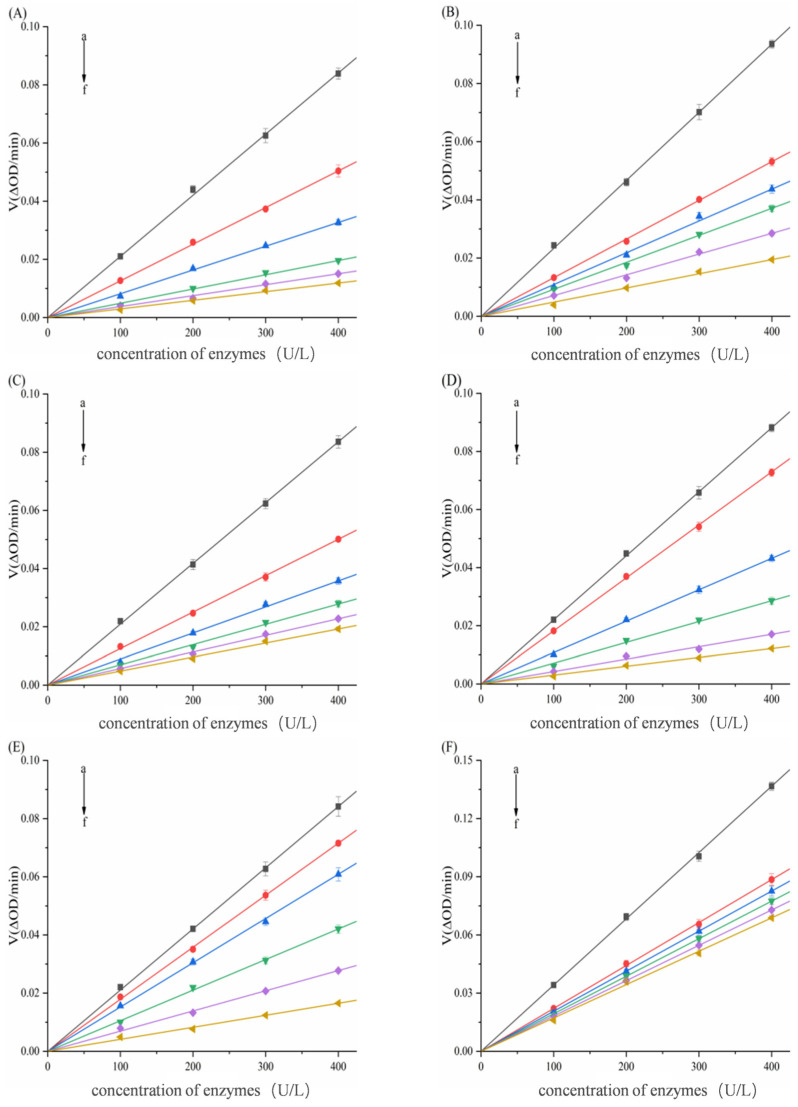
Analysis of reversible inhibition of α-glucosidase by six porphyrins. (**A**) THPP, (**B**) Ni–TCPP, (**C**) Fe–TCPP, (**D**) TCPP, (**E**) Cu–TCPP, (**F**) Tapp’s reversibility of binding to α-glucosidase a→f is (**A**) c (THPP) = 0, 1, 2.5, 5, 7.5, 10 μg/mL; (**B**) c (Ni–TCPP) = 0, 5, 7.5, 10, 15, 25 μg/mL; (**C**) c (Fe–TCPP) = 0, 16, 32, 48, 64, 80 μg/mL; (**D**) c (TCPP) = 0, 5, 25, 50, 100, 150 μg/mL; (**E**) c (Cu–TCPP) = 0, 5, 10, 25, 50, 100 μg/mL; (**F**) c(TAPP) = 0, 12.5, 15, 17.5, 20, 22.5 μg/mL.

In Figure 4 and Figure 5, despite structural differences and varying concentrations among the six compounds, all linear plots intersected the origin. The slope of these lines decreased with increasing porphyrin concentration, demonstrating reversible inhibition of α-amylase/α-glucosidase activity by TAPP, THPP, TCPP, Ni–TCPP, Cu–TCPP, and Fe–TCPP.

**Figure 6:**
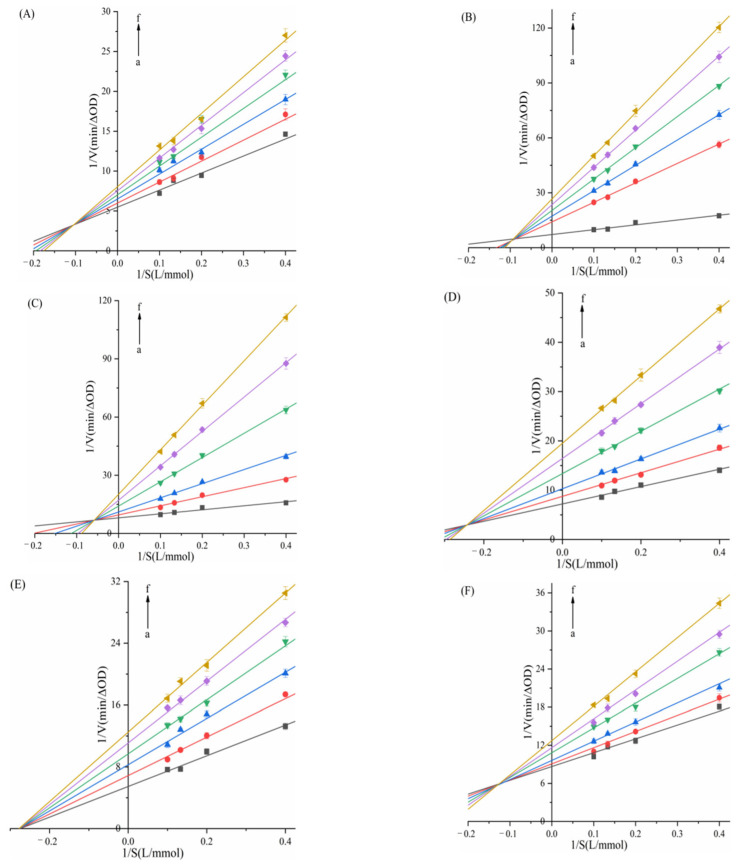
Lineweaver–Burk double-reciprocal curve of (**A**) TAPP, (**B**) THPP, (**C**) TCPP, (**D**) Fe–TCPP, (**E**) Ni–TCPP, (**F**) Cu–TCPP for α-amylase (a→f, ibid., Figure 4).

**Figure 7:**
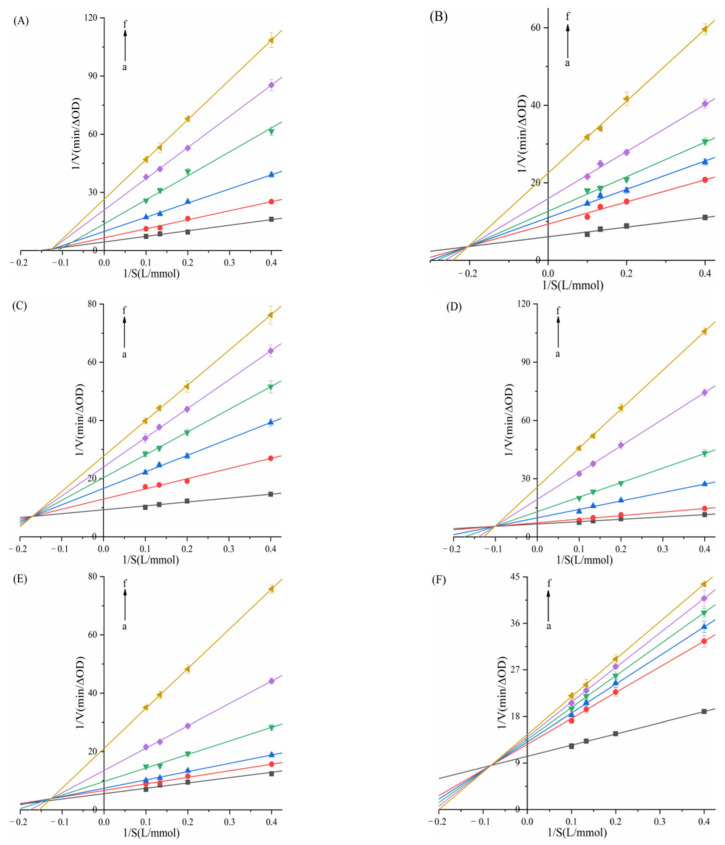
Lineweaver–Burk double-reciprocal curves of (**A**) THPP, (**B**) Ni–TCPP, (**C**) Fe–TCPP, (**D**) TCPP, (**E**) Cu–TCPP, (**F**) TAPP for α-glucosidase (a→f, same as Figure 5 above).

To further investigate the types of inhibition of α-amylase/α-glucosidase by six porphyrins. Using Lineweaver–Burk double-reciprocal plots, with the reciprocal of reaction velocity (1/V) as the ordinate and the reciprocal of substrate concentration (1/S) as the abscissa, as shown in Figure 6 and Figure 7. For α-amylase (Figure 6), the Lineweaver–Burk plots of TAPP, THPP, TCPP, Cu–TCPP, and Fe–TCPP intersected in the second quadrant, and it can be concluded that the inhibition is a mixed type (competitive–non-competitive). In contrast, the double-reciprocal plots for Ni–TCPP show lines converging on the *x*-axis, indicating non-competitive inhibition. For α-glucosidase (Figure 7), the straight line for THPP intersects the *x*-axis, indicating non-competitive inhibition. For Ni–TCPP, Fe–TCPP, TCPP, Cu–TCPP, and TAPP, the lines intersect in the second quadrant, and it can be concluded that the inhibition is a mixed type.

*K_i_* and *K_is_* are calculated using Formulas (4) and (5) and are presented in Table 1 and Table 2. For α-amylase (Table 1), increasing concentrations of the five porphyrin compounds progressively elevated the *K_m_* values while reducing *V*_max_. Therefore, the inhibition of α-amylase by TAPP, THPP, TCPP, Fe–TCPP, and Cu–TCPP is classified as mixed inhibition. *K_m_* remains essentially unchanged, while *V*_max_ progressively decreases with increasing Ni–TCPP concentration, demonstrating that this is typical non-competitive inhibition. Table 1 also shows that *K_i_* for five porphyrin compounds is lower than their respective *K_is_* values. This indicates that TAPP, THPP, TCPP, Fe–TCPP, and Cu–TCPP exhibit stronger binding affinity to the free α-AMY than to the enzyme–substrate complex. The calculated *K_i_* values were in the order of α-AMY–TAPP (*K_i_* = 8.7057 ± 0.0325 μg/mL) > α-AMY–THPP (*K_i_* = 9.2445 ± 0.0411 μg/mL) > α-AMY–TCPP (*K_i_* = 19.9216 ± 0.0524 μg/mL) > α-AMY–Fe–TCPP (*K_i_* = 69.5865 ± 0.1284 μg/mL) > α-AMY–Cu–TCPP (*K_i_* = 135.8043 ± 0.1608 μg/mL). A smaller *K_i_* value indicates stronger inhibitory activity [43,44]. Therefore, the order of inhibitory ability of the porphyrin compounds against α-amylase is TAPP > THPP > TCPP > Fe–TCPP > Cu–TCPP, which is consistent with the conclusion drawn from the IC_50_ values.

For α-glucosidase, data in Table 2 demonstrate that with increasing THPP concentration, the *K_m_* value remains essentially constant, while *V*_max_ gradually decreases. Thus, THPP exerts non-competitive inhibition on α-glucosidase and indicates that it does not affect the affinity of the substrate with α-glucosidase. Table 2 also shows that increasing concentrations of these five porphyrin compounds result in progressive increases in *K_m_* and decreases in *V*_max_, confirming that their inhibition of α-glucosidase follows a mixed mechanism. For five porphyrin compounds, *K_i_* < *K_is_* indicates stronger binding affinity to free α-GLU than to the enzyme–substrate complex. The calculated *K_i_* values were in the order of α-GLU–THPP (*K_i_* = 1.6508 ± 0.0135 μg/mL) > α-GLU–Ni–TCPP (*K_i_* = 3.8703 ± 0.0201 μg/mL) > α-GLU–TAPP (*K_i_* = 9.4635 ± 0.0572 μg/mL) > α-GLU–TCPP (*K_i_* = 9.5746 ± 0.0611 μg/mL) > α-GLU–Fe–TCPP (*K_i_* = 9.8917 ± 0.0514 μg/mL) > α-GLU–Cu–TCPP (*K_i_* = 13.7332 ± 0.0823 μg/mL). Therefore, the order of inhibitory ability of the porphyrin compounds against α-glucosidase is THPP > Ni–TCPP > Fe–TCPP > TCPP > Cu–TCPP > TAPP.

In summary, based on a comprehensive analysis of the IC_50_ values and inhibition constants, THPP is an effective inhibitor that exhibits potent inhibitory effects against both enzymes.

### 3.3. Study of Stability In Vitro

**Figure 8:**
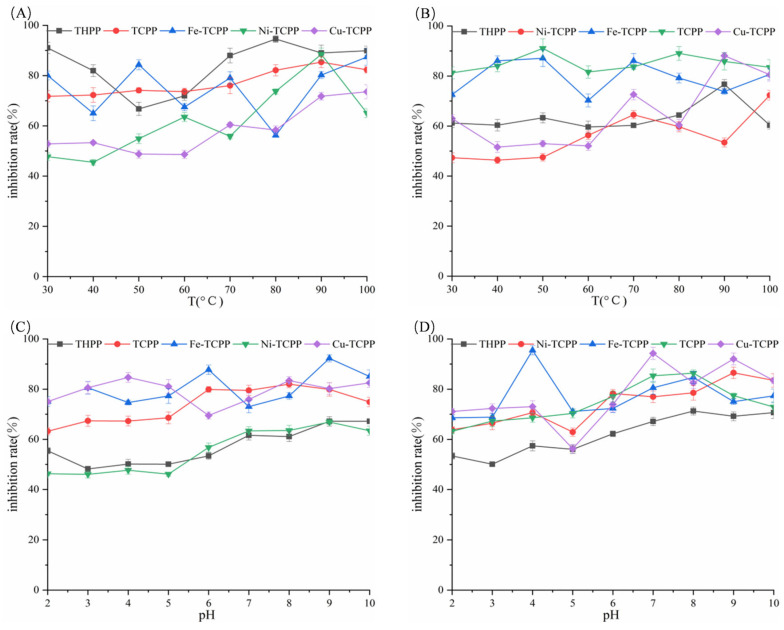
In vitro stability study curves. (**A**,**B**) Temperature. (**C**,**D**) Acid–base stability.

As shown in Figure 8A, THPP, TCPP, Fe–TCPP, Ni–TCPP, and Cu–TCPP exhibited small fluctuations in their inhibition rates against α-amylase within the temperature range of 30–100 °C. Similarly, as illustrated in Figure 8B, the inhibition rate curves of THPP, Ni–TCPP, Fe–TCPP, TCPP, and Cu–TCPP against α-glucosidase remained relatively stable, indicating that these porphyrin compounds possess good thermal stability over short periods. As depicted in Figure 8C,D, the porphyrin compounds demonstrated a broad pH tolerance range (pH 2.0–10.0). Furthermore, they exhibited stronger inhibitory effects under weakly alkaline conditions compared to acidic conditions. The systems that were not subjected to in vitro stability studies were excluded because their inhibition rates against both enzymes failed to reach the threshold of 60–90% set for this study within the experimental concentration range.

### 3.4. Fluorescence Quenching and Binding of Enzymes by Six Porphyrin Compounds

**Figure 9:**
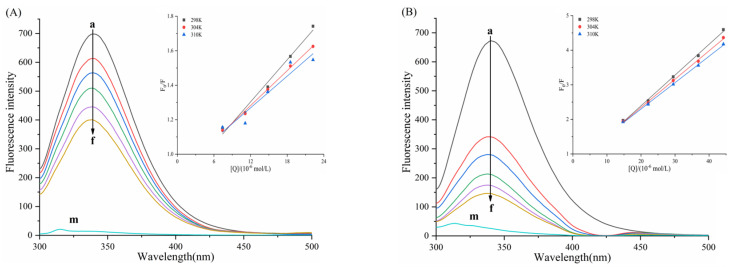

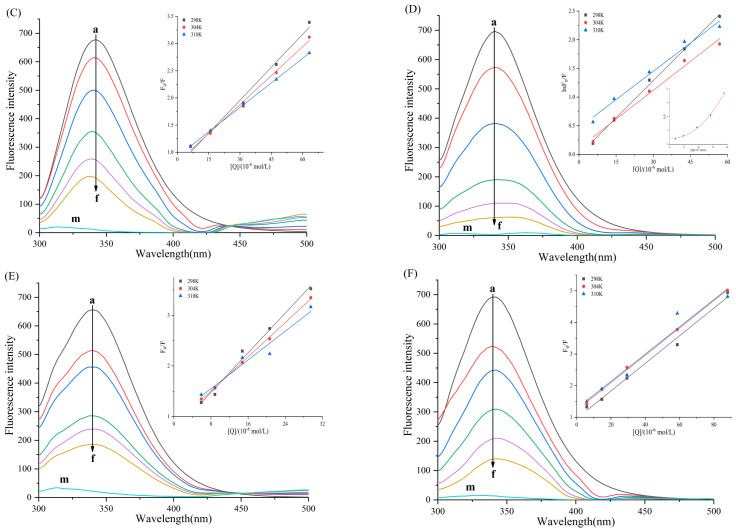
Fluorescence spectra of (**A**) TAPP, (**B**) THPP, (**C**) TCPP, (**D**) Fe–TCPP, (**E**) Ni–TCPP, (**F**) Cu–TCPP for α-amylase a→f are (**A**) c (TAPP) = 0, 5, 7.5, 10, 12.5, 15 μg/mL; (**B**) c (THPP) = 0, 10, 15, 20, 25, 30 μg/mL; (**C**) c (TCPP) = 0, 5, 12.5, 25, 37.5, 50 μg/mL; (**D**) c (Fe–TCPP) = 0, 5, 12.5, 25, 37.5, 50 μg/mL; (**E**) c (Ni–TCPP) = 0, 5, 7.5, 12.5, 17.5, 25 μg/mL; (**F**) c (Cu–TCPP) = 0, 5, 12.5, 25, 50, 75 μg/mL m shows only the emission spectra of small molecules.

**Figure 10:**
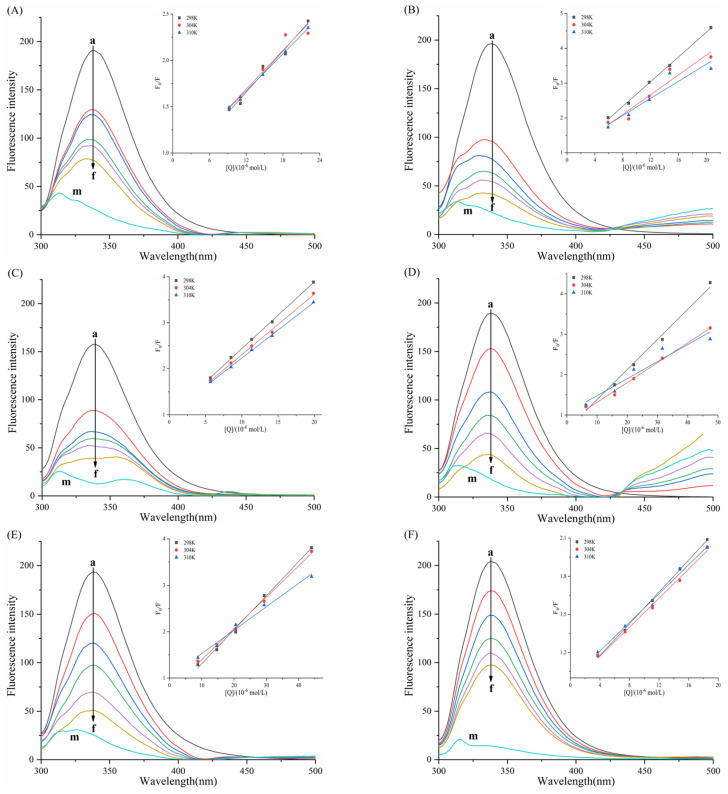
(**A**) THPP, (**B**) Ni–TCPP, (**C**) Fe–TCPP, (**D**) TCPP, (**E**) Cu–TCPP, (**F**) TAPP fluorescence spectra of α-glucosidase a→f are (**A**) c (THPP) = 0, 6.25, 7.5, 10, 12.5, 15 μg/mL; (**B**) c (Ni–TCPP) = 0, 5, 7.5, 10, 15, 17.5 μg/mL; (**C**) c (Fe–TCPP) = 0, 5, 7.5, 10, 12.5, 17.5 μg/mL; (**D**) c (TCPP) = 0, 5, 12.5, 17.5, 25, 37.5 μg/mL; (**E**) c (Cu–TCPP) = 0, 7.5, 12.5, 17.5, 25, 37.5 μg/mL; (**F**) c (TAPP) = 0, 2.5, 5, 7.5, 10, 12.5 μg/mL m, where only the emission spectra of small molecules are shown.

As shown in the fluorescence spectra of the six porphyrin compounds with α-amylase/α-glucosidase (Figure 9 and Figure 10), the fluorescence intensity of both enzymes gradually decreased with the addition of the compounds, indicating the presence of an interaction that quenched the intrinsic fluorescence of the enzymes.

For α-amylase (Figure 9), with increasing concentrations of TAPP, THPP, and TCPP, the emission wavelength of α-amylase exhibites a blue shift. The magnitude of this shift followed the order TCPP > THPP > TAPP, resulting in reduced polarity and enhancing the hydrophobicity of the microenvironment surrounding amino acid residues in α-amylase. This phenomenon is typically associated with protein folding. Conversely, Fe–TCPP and Cu–TCPP induced a red shift in the emission wavelength at higher concentrations. This signifies alterations in the microenvironments of α-amylase residues. Compared to Cu–TCPP, Fe–TCPP caused a more pronounced increase in polarity and a greater reduction in hydrophobicity, suggesting a greater propensity to induce structural unfolding of the protein [45].

For α-glucosidase (Figure 10), with increasing concentrations of THPP, Ni–TCPP, Fe–TCPP, TCPP, and Cu–TCPP, a blue shift in the emission maximum of α-glucosidase occurred. The magnitude of the blue shift followed the order Ni–TCPP > THPP > Fe–TCPP > TCPP > Cu–TCPP.

**Figure 11:**
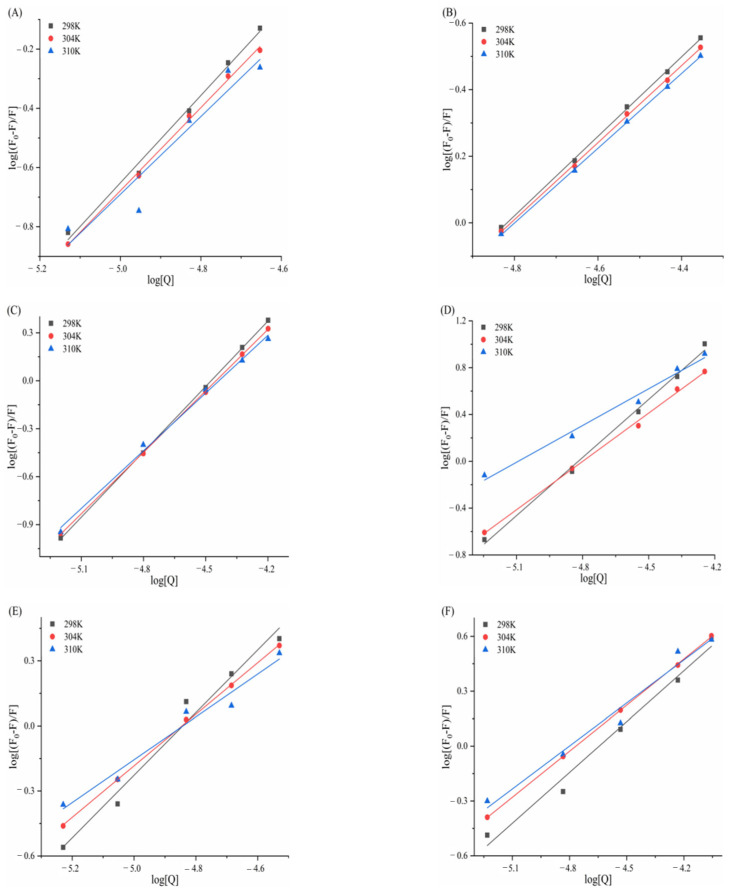
(**A**) TAPP, (**B**) THPP, (**C**) TCPP, (**D**) Fe–TCPP, (**E**) Ni–TCPP, (**F**) Cu–TCPP double logarithmic curves for α-amylase.

In Figure 11, linear Stern–Volmer plots were observed for TAPP, THPP, TCPP, Ni–TCPP, and Cu–TCPP. Table 3 shows decreasing *K_SV_* values with rising temperature and *K_q_* values substantially exceeding 2.0 × 10^10^ L/mol/s, indicating pure static quenching of α-amylase by these five porphyrins. For the α-amylase–Fe–TCPP system, the Stern–Volmer plot exhibited upward curvature (positive deviation toward the *y*-axis), signifying mixed quenching behavior. Table 3 reveals an inverse temperature dependence of *K_SV_* and *K_q_* > 2.0 × 10^10^ M^−1^s^−1^, confirming a hybrid quenching mechanism where static quenching prevails over dynamic processes in the Fe–TCPP–α-amylase system. At 298, 304, and 310K, linear correlations between log[(*F*_0_ − *F*)/*F*] and log[*Q*] were observed for all six porphyrins. K_a_ and *n* can be calculated from the intercept and slope of the curve, respectively. Table 3 indicates *n* ≈ 1, confirming a single binding site on α-amylase for each compound.

K_a(Cu-TCPP)_ = 10^3^–10^4^ L/mol, displaying moderate binding affinity. The other five porphyrins exhibited stronger binding capabilities. Decreasing *K_a_* with rising temperature reflects reduced stability of the porphyrin–α-amylase complexes. At 310 K, the binding constants followed the order TAPP > THPP > TCPP > Fe–TCPP > Ni–TCPP > Cu–TCPP; this sequence aligns with their binding affinities and inhibitory potencies.

**Figure 12:**
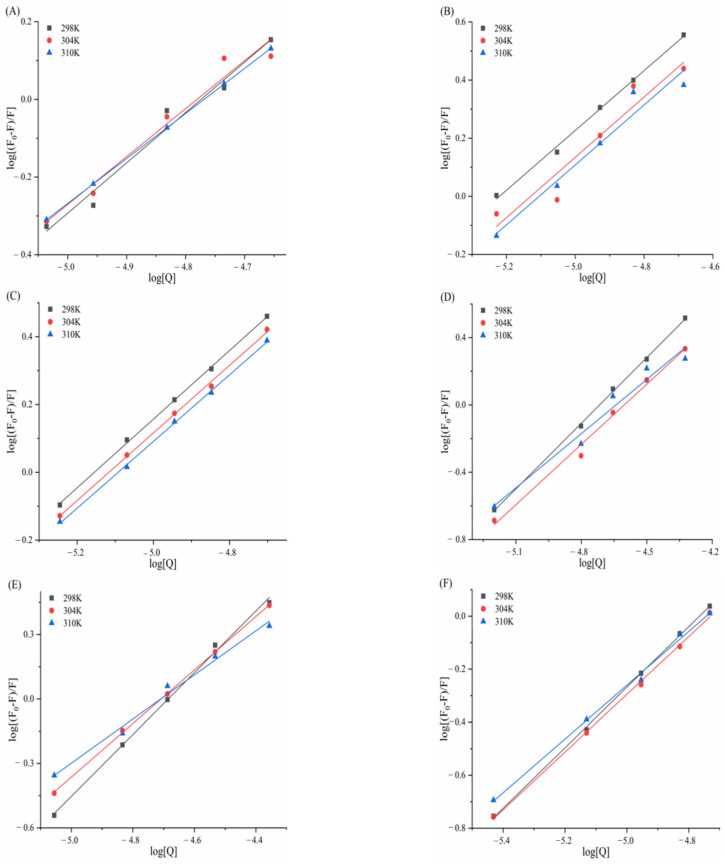
(**A**) THPP, (**B**) Ni–TCPP, (**C**) Fe–TCPP, (**D**) TCPP, (**E**) Cu–TCPP, (**F**) TAPP double logarithmic curves for α-GLU.

Figure 12 reveals linear Stern–Volmer plots for all six porphyrin compounds. Table 4 reveals an inverse temperature dependence of *K_SV_* and *K_q_* (>>2.0 × 10^10^ M^−1^s^−1^), confirming that the quenching mechanism of THPP, Ni–TCPP, Fe–TCPP, TCPP, Cu–TCPP, and TAPP on α-glucosidase was static quenching. Figure 12 displays linear correlations between log[(*F*_0_ − *F*)/*F*] and log[*Q*] at 298, 304, and 310 K. Table 4 indicates *n* ≈ 1, confirming a single binding site on *α*-glucosidase for each compound. All porphyrins exhibit favorable binding properties to *α*-GLU. Moreover, increasing temperature results in destabilization of the porphyrin–α-glucosidase complexes.

At 310 K, the binding constants K of the six porphyrin-based compounds were determined as THPP > Ni–TCPP > Fe–TCPP > TCPP > Cu–TCPP > TAPP, with corresponding values of (3.39 ± 0.03) × 10^5^, (1.83 ± 0.01) × 10^5^, (1.02 ± 0.02) × 10^5^, (0.97 ± 0.02) × 10^5^, (0.69 ± 0.01) × 10^5^, and (0.65 ± 0.02) × 10^5^ L·mol^−1^. A larger binding constant and a smaller IC_50_ value indicate stronger affinity of the inhibitor. Consequently, the inhibitory potency of the six porphyrin compounds against α-glucosidase were ranked as THPP > Ni–TCPP > Fe–TCPP > TCPP > Cu–TCPP > TAPP.

### 3.5. Determination of Thermodynamic Parameters and Main Forces

**Figure 13:**
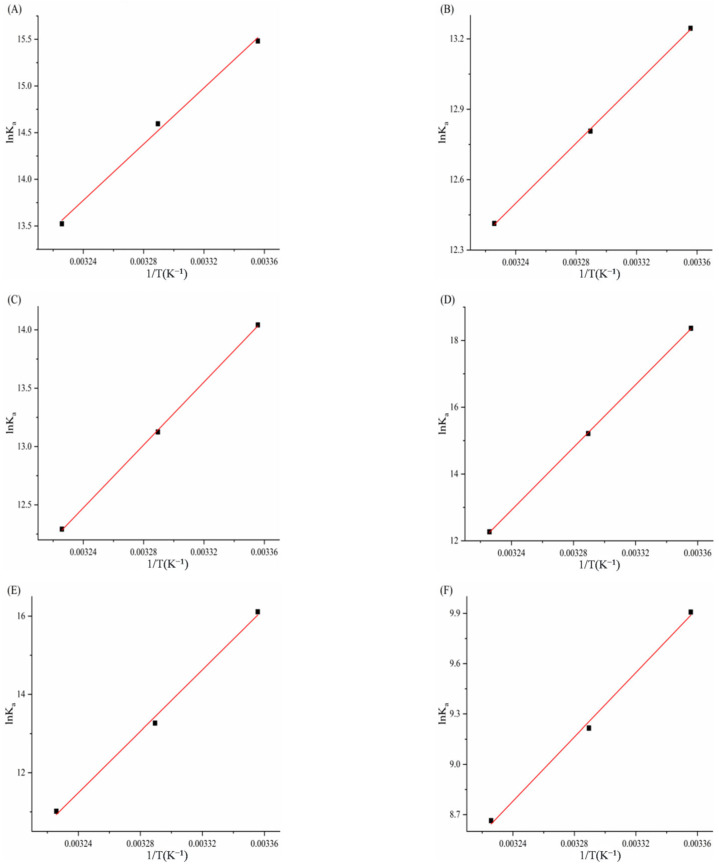
(**A**) TAPP, (**B**) THPP, (**C**) TCPP, (**D**) Fe–TCPP, (**E**) Ni–TCPP, (**F**) Cu–TCPP Van’t Hoff curve for α-amylase.

**Figure 14:**
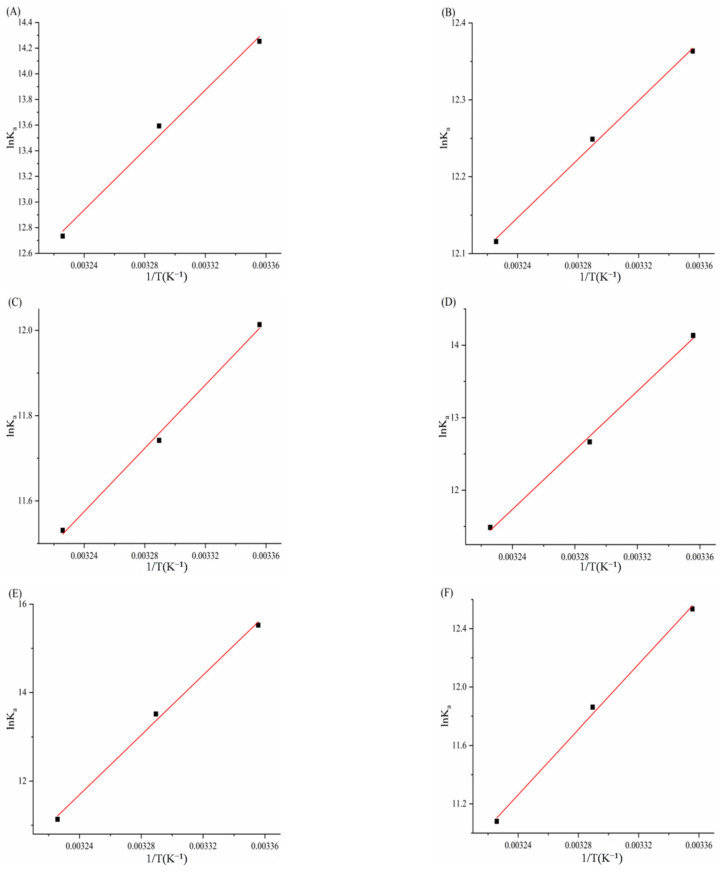
(**A**) THPP, (**B**) Ni–TCPP, (**C**) Fe–TCPP, (**D**) TCPP, (**E**) Cu–TCPP, (**F**) TAPP Van’t Hoff curve for α-glucosidase.

Figure 13 and Figure 14 display Van’t Hoff plots of ln*Kₐ* against 1/*T* for the six porphyrin compounds with α-amylase/α-glucosidase, and the thermodynamic parameters calculated using Formula 9 and 10 are presented in Table 5 and Table 6. Negative Gibbs free energy changes (ΔG^0^ < 0) for all compounds confirm that the interactions between α-amylase/α-glucosidase and TAPP, THPP, TCPP, Fe–TCPP, Ni–TCPP, and Cu–TCPP are spontaneous processes. The observed negative enthalpy changes (ΔH^0^ < 0) and negative entropy changes (ΔS^0^ < 0) during complex formation indicate that binding is predominantly driven by hydrogen bonding and van der Waals forces.

In Table 6, for the complexes of α-glucosidase with THPP, Fe–TCPP, TCPP, Cu–TCPP, and TAPP, both negative enthalpy changes (ΔH^0^ < 0) and negative entropy changes (ΔS^0^ < 0) were observed, suggesting that binding is primarily driven by hydrogen bonding and van der Waals forces. In contrast, the α-glucosidase–Ni–TCPP complex exhibited a negative enthalpy change (ΔH^0^ < 0) but a positive entropy change (ΔS^0^ > 0), indicating electrostatic interactions as the dominant binding force.

### 3.6. Effect of Porphyrins on the Synchronized Fluorescence Spectra

**Figure 15:**
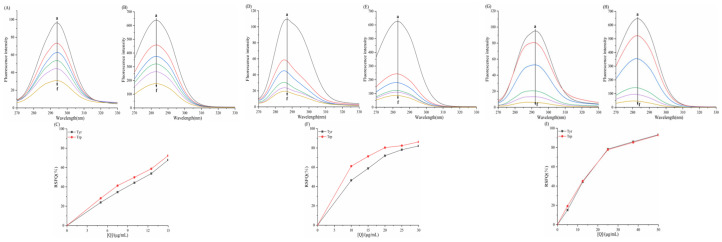
Synchronous fluorescence spectroscopy TAPP for α-amylase: (**A**) Δλ = 15 nm; (**B**) Δλ = 60 nm; (**C**) RSFQ THPP for α-amylase; (**D**) Δλ = 15 nm; (**E**) Δλ = 60 nm; (**F**) RSFQ TCPP for α-amylase; (**G**) Δλ = 15 nm; (**H**) Δλ = 60 nm; (**I**) RSFQ. The labels a→f correspond to the following concentrations: (**A**) c(TAPP) = 0, 5, 7.5, 10, 12.5, 15 μg/mL; (**B**) c(THPP) = 0, 10, 15, 20, 25, 30 μg/mL; (**C**) c(TCPP) = 0, 5, 12.5, 25, 37.5, 50 μg/mL; (**D**) c(Fe-TCPP) = 0, 5, 12.5, 25, 37.5, 50 μg/mL; (**E**) c(Ni-TCPP) = 0, 5, 7.5, 12.5, 17.5, 25 μg/mL; (**F**) c(Cu-TCPP) = 0, 5, 12.5, 25, 50, 75 μg/mL.

**Figure 16:**
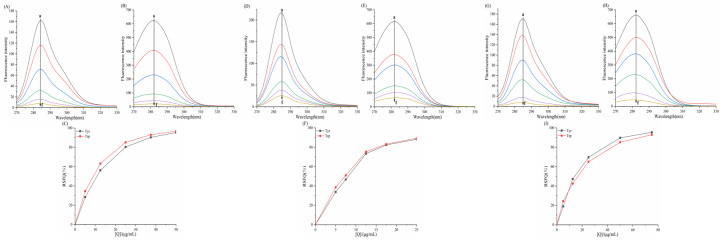
Synchronous fluorescence spectroscopy Fe–TCPP for α-amylase: (**A**) Δλ = 15 nm; (**B**) Δλ = 60 nm; (**C**) RSFQ Ni–TCPP for α-amylase; (**D**) Δλ = 15 nm; (**E**) Δλ = 60 nm; (**F**) RSFQ Cu–TCPP for α-amylase; (**G**) Δλ = 15 nm; (**H**) Δλ = 60 nm (**I**) RSFQ. a→f corresponds to the same concentrations as in Figure 15.

**Figure 17:**
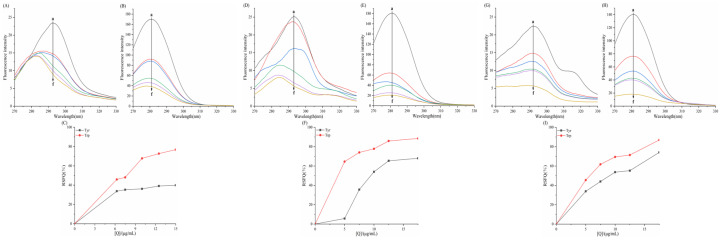
Synchronous fluorescence spectroscopy THPP for α-glucosidase: (**A**) Δλ = 15 nm; (**B**) Δλ = 60 nm; (**C**) RSFQ Ni–TCPP for α-glucosidase; (**D**) Δλ = 15 nm; (**E**) Δλ = 60 nm; (**F**) RSFQ Fe–TCPP for α-glucosidase; (**G**) Δλ = 15 nm; (**H**) Δλ = 60 nm; (**I**) RSFQ. a→f corresponds to the same concentrations as in Figure 15.

**Figure 18:**
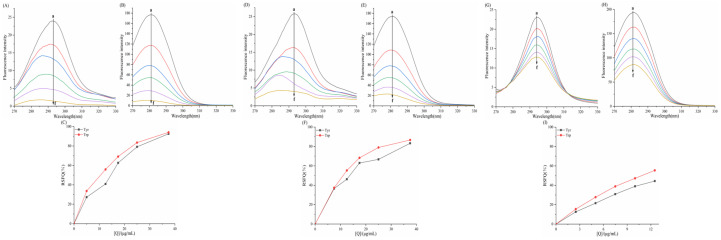
Synchronous fluorescence spectroscopy TCPP for α-glucosidase: (**A**) Δλ = 15 nm; (**B**) Δλ = 60 nm; (**C**) RSFQ; Cu–TCPP for α-glucosidase; (**D**) Δλ = 15 nm; (**E**) Δλ = 60 nm; (**F**) RSFQ; TAPP for α-glucosidase; (**G**) Δλ = 15 nm; (**H**) Δλ = 60 nm; (**I**) RSFQ. a→f corresponds to the same concentrations as in Figure 15.

In Figure 15, Figure 16, Figure 17 and Figure 18 the fluorescence intensity of tyrosine (Tyr) and tryptophan (Trp) residues in the system exhibits a negative correlation with the concentration of porphyrin compounds, indicating interactions between α-amylase/α-glucosidase and TAPP, THPP, TCPP, Fe–TCPP, Ni–TCPP, and Cu–TCPP.

#### 3.6.1. Effect of Porphyrins on the Synchronized Fluorescence Spectra of α-AMY

In the α-amylase–TAPP system, the maximum emission wavelength of tyrosine and tryptophan residues in the α-amylase–TAPP system showed no significant shift. This indicates that TAPP essentially does not alter the microenvironment of Tyr and Trp within α-amylase, exerting only a minimal effect on the polarity and hydrophobicity surrounding these residues. For the α-amylase–THPP system, a slight blue shift occurred in the Tyr emission maximum while the Trp peak remained unchanged, suggesting reduced polarity and increased hydrophobicity around Tyr residues but no significant Trp perturbation. Increasing TCPP concentrations induced blue shifts in both Tyr and Trp emission maxima, signifying decreased polarity and increased hydrophobicity around these residues. With increasing Fe–TCPP concentrations, Tyr synchronous fluorescence showed a blue shift but Trp exhibited a red shift, indicating reduced polarity/hydrophobicity around Tyr residues versus increased polarity/decreased hydrophobicity around Trp residues (implying greater Trp solvent exposure). For Ni–TCPP, no Tyr spectral shift occurred, but Trp showed a red shift, demonstrating minimal Tyr effects but increased polarity/reduced hydrophobicity around Trp. Conversely, Cu–TCPP induced a Trp blue shift without altering Tyr spectra, indicating decreased polarity/increased hydrophobicity specifically around Trp residues.

As depicted in Figure 15 and Figure 16, the relative synchronous fluorescence quenching (RSFQ) values at tryptophan (Trp) residues exceeded those at tyrosine (Tyr) residues for TAPP, THPP, Fe–TCPP, and Ni–TCPP. This indicates a greater contribution of Trp residues to the intrinsic fluorescence quenching of α-amylase by these compounds, suggesting their closer proximity to Trp sites. Conversely, TCPP showed comparable RSFQ values at both residues, demonstrating simultaneous binding near Tyr and Trp residues. Distinctively, Cu–TCPP exhibited higher quenching efficiency at Tyr residues, signifying the predominant contribution of Tyr residues and preferential localization near Tyr sites.

#### 3.6.2. Effect of Porphyrins on the Synchronized Fluorescence Spectra of α-GLU

For THPP, Ni–TCPP, Fe–TCPP, TCPP, and Cu–TCPP, concentration-dependent blue shifts in both Tyr and Trp emission maxima demonstrated decreased polarity and enhanced hydrophobicity around these residues during enzyme binding. In contrast, the α-glucosidase–TAPP system exhibited only a slight Tyr blue shift with unaltered Trp emission, indicating a localized reduction in Tyr polarity/hydrophobicity and minimal Trp perturbation.

In Figure 17 and Figure 18, all six porphyrin-based compounds exhibited consistently higher relative synchronous fluorescence quenching (RSFQ) values at tryptophan residues compared to tyrosine residues. This indicates that in the fluorescence quenching of α-glucosidase by THPP, Ni–TCPP, Fe–TCPP, TCPP, Cu–TCPP, and TAPP, tryptophan (Trp) residues contribute predominantly, suggesting that the quenching sites are in closer proximity to Trp residues.

### 3.7. Effect of Porphyrins on the Three-Dimensional Fluorescence Spectra of Enzymes

Fluorescence peak 1 primarily reveals the spectral signature of Trp and Tyr residues, and peak 2 represents changes in peptide chain conformation.

#### 3.7.1. Effect of Porphyrins on the Three-Dimensional Fluorescence Spectra of α-Amylase

**Figure 19:**
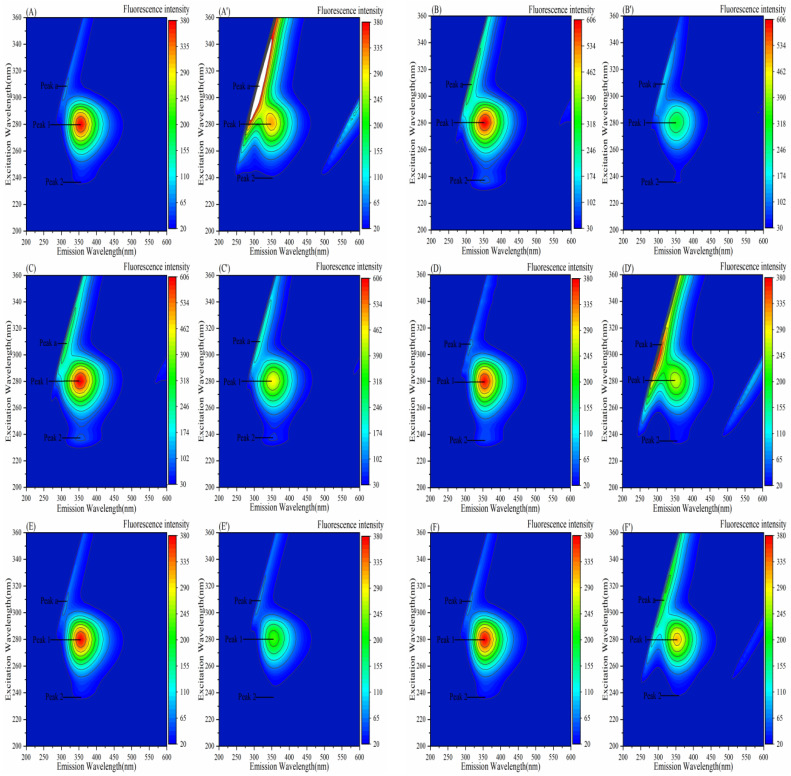
Three-dimensional fluorescence spectra of porphyrin interactions with α-AMY: (**A**−**F**) α-AMY; (**A’**−**F’**) α-AMY–TAPP, α-AMY–THPP, α-AMY–TCPP, α-AMY–Fe–TCPP, α-AMY–Ni–TCPP, α-AMY–Cu–TCPP; c (TAPP) = 2.5 μg/mL, c (THPP) = 10 μg/mL, c (TCPP) = 10 μg/mL, c (Fe–TCPP) = 12.5 μg/mL, c (Ni–TCPP) = 17.5 μg/mL, c (Cu–TCPP) = 10 μg/mL.

Figure 19 and Table 7 demonstrate decreased fluorescence intensities at peaks 1 and 2 for all six porphyrin-based compounds, indicating Tyr/Trp fluorescence quenching and α-amylase conformational changes. Peak 1 reduction likely stems from attenuated intrinsic emission in enzyme–porphyrin complexes, while decreased peak 2 intensity suggests polypeptide destabilization and altered hydrophobicity inducing structural reorganization. Notably, peak a intensity increased with TAPP/Fe–TCPP/Cu–TCPP due to enhanced light scattering from enlarged protein complexes. Conversely, THPP and TCPP reduced peak a intensity, potentially through hydration layer disruption and surface-binding-induced disaggregation, decreasing particle size and scattering. This is consistent with the results of synchronous fluorescence spectroscopy analysis.

#### 3.7.2. Effect of Porphyrins on the Three-Dimensional Fluorescence Spectra of α-Glucosidase

**Figure 20:**
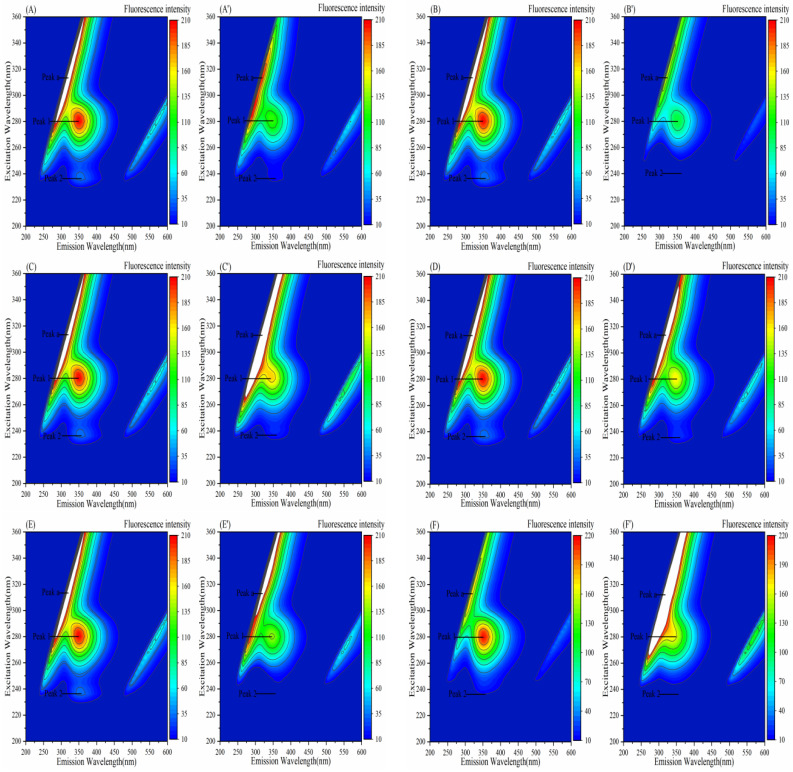
Three-dimensional fluorescence spectra of porphyrins interacting with α-GLU: (**A**−**F**) α-GLU; (**A’**−**F’**) α-GLU–THPP, α-GLU–Ni–TCPP, α-GLU–Fe–TCPP, α-GLU–TCPP, α-GLU–Cu–TCPP, α-GLU–TAPP; c (THPP) = 7.5 μg/mL, c (Ni–TCPP) = 10 μg/mL, c (Fe–TCPP) = 7.5 μg/mL, c (TCPP) = 10 μg/mL, c (Cu–TCPP) = 17.5 μg/mL, c (TAPP) = 7.5 μg/mL.

In Figure 20 and Table 8, the fluorescence intensities of peak 1 and peak 2 decreased upon the addition of the six porphyrin compounds, with peak 2 for TAPP nearly disappearing. This indicates that the addition of these porphyrins likely quenched the fluorescence emission from the Tyr and Trp residues of α-glucosidase and induced the unfolding of the α-glucosidase polypeptide. Furthermore, the intensity change of peak 2 for TAPP was significantly greater than that of peak 1, suggesting that conformational changes play a more significant role in the formation of the α-glucosidase–TAPP complex. The formation of the α-glucosidase–Fe–TCPP and α-glucosidase–TAPP complexes likely increased the protein diameter, leading to an increase in the fluorescence intensity of peak a. In contrast, the added THPP and Ni–TCPP may have disrupted the hydration layer upon binding to the surface of α-glucosidase, resulting in a decrease in protein diameter and a concomitant reduction in the fluorescence intensity of peak a.

### 3.8. Effect of Porphyrins on the Infrared Spectrum of Enzymes

#### 3.8.1. Effect of Porphyrins on the Infrared Spectrum of α-Amylase

For proteins, Fourier transform infrared (FTIR) spectroscopy provides information on amino group vibrations, where the amide I band (C=O stretching) and amide II band (N-H bending or C-N stretching) are particularly relevant to this study. These bands correspond to wavenumber ranges of 1700–1600 cm^−1^ and 1530–1550 cm^−1^, respectively. To quantify the secondary structural composition, curve-fitting analysis was applied to deconvolute these bands into sub-peaks, with each sub-peak assigned to one of five protein conformations: α-helix (1649–1660 cm^−1^), β-sheet (1615–1637 cm^−1^), β-turn (1661–1680 cm^−1^), random coil (1681–1692 cm^−1^), and antiparallel β-sheet (1638–1648 cm^−1^) [46].

**Figure 21:**
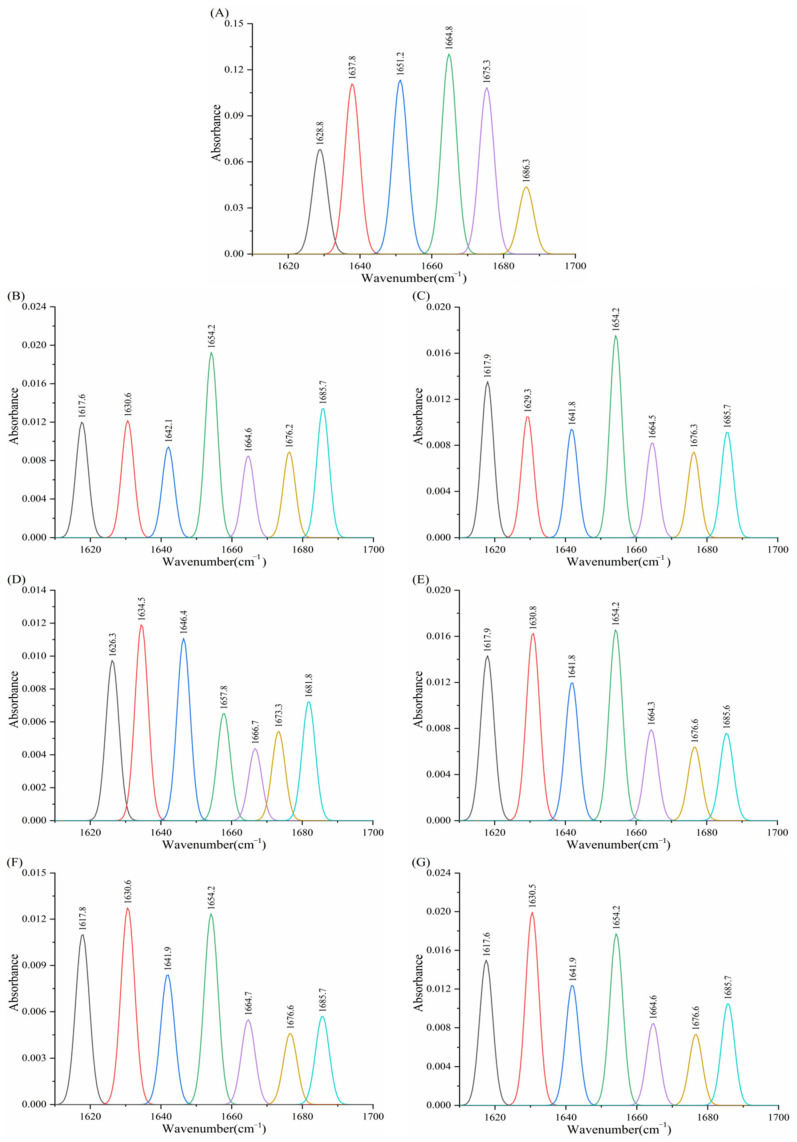
(**A**) α-AMY, (**B**) α-AMY–TAPP, (**C**) α-AMY–THPP, (**D**) α-AMY–TCPP, (**E**) α-AMY–Fe–TCPP, (**F**) α-AMY–Ni–TCPP, (**G**) α-AMY–Cu–TCPP FT-IR spectral amide I.

Deconvolution of the amide I band in the FT-IR spectra of α-amylase and its complexes with porphyrin compounds yielded Figure 21, with the calculated percentages of each secondary structural component presented in Table 9. The results show that native α-amylase comprises 24.58% α-helix, 27.34% β-sheet, 23.64% β-turn, 14.10% random coil, and 10.34% β-antiparallel structures. Upon the addition of TAPP, THPP, TCPP, Fe–TCPP, Ni–TCPP, and Cu–TCPP, the proportions of α-helix and β-turn decreased, while β-sheet content increased. Specifically, TAPP reduced the random coil content to 9.94% and increased β-antiparallel structures to 18.98%. In contrast, the other five porphyrin compounds increased random coil content. Among these, THPP, TCPP, and Cu–TCPP elevated β-antiparallel structures, whereas Fe–TCPP and Ni–TCPP reduced its proportion.

The above results demonstrate that TAPP, THPP, TCPP, Ni–TCPP, Cu–TCPP, and Fe–TCPP induce alterations in the secondary structure of α-amylase. Given that hydrogen bonds maintain the stability of protein secondary structures, the observed structural changes suggest potential disruption of the hydrogen-bonding network within α-amylase. This destabilizes the enzyme’s conformation, resulting in structural flexibility and instability. Consequently, it may impair the formation of the active site and/or hinder substrate access to the catalytic center, ultimately reducing α-amylase activity.

#### 3.8.2. Effect of Porphyrins on the Infrared Spectroscopy of α-Glucosidase

**Figure 22:**
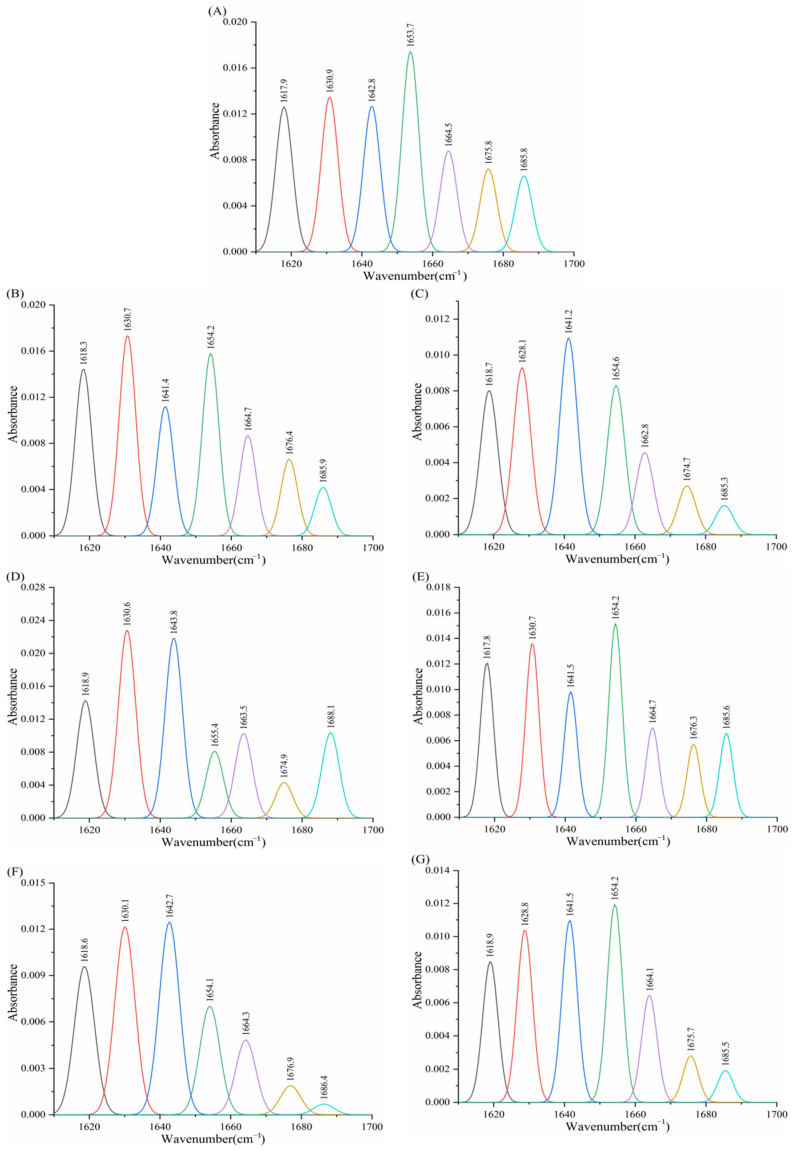
(**A**) α-glucosidase, (**B**) α-glucosidase–THPP, (**C**) α-glucosidase–Ni–TCPP, (**D**) α-glucosidase–Fe–TCPP, (**E**) α-glucosidase–TCPP, (**F**) α-glucosidase–Cu–TCPP, (**G**) α-glucosidase–TAPP FT-IR spectral amide I band fitting diagram.

In Figure 22 and Table 10, native α-glucosidase consists of 24.74% α-helix, 32.91% β-sheet, 14.72% β-turn, 19.42% random coil, and 8.21% β-antiparallel structures. Following the addition of the six porphyrin compounds, α-helix content decreased while β-sheet content increased. Specifically, Fe–TCPP elevated β-turn’s proportion to 15.77%, reduced random coil to 16.75%, and decreased β-antiparallel structures to 8.02%. In contrast, the other five compounds diminished β-turn content and increased random coil. Among them, THPP, Ni–TCPP, Cu–TCPP, and TAPP reduced β-antiparallel structures, whereas TCPP increased its proportion. These findings indicate that binding of THPP, Ni–TCPP, Fe–TCPP, TCPP, Cu–TCPP, and TAPP to amino acid residues of α-glucosidase likely disrupts the hydrogen-bonding network, inducing partial protein unfolding and altering active site conformation. Concurrently, the secondary structure undergoes restructuring toward disorder, impairing substrate binding and ultimately diminishing enzymatic activity.

### 3.9. Molecular Docking Analysis of Porphyrins to Enzymes

#### 3.9.1. Molecular Docking Analysis of Porphyrins with α-Amylase

**Figure 23:**
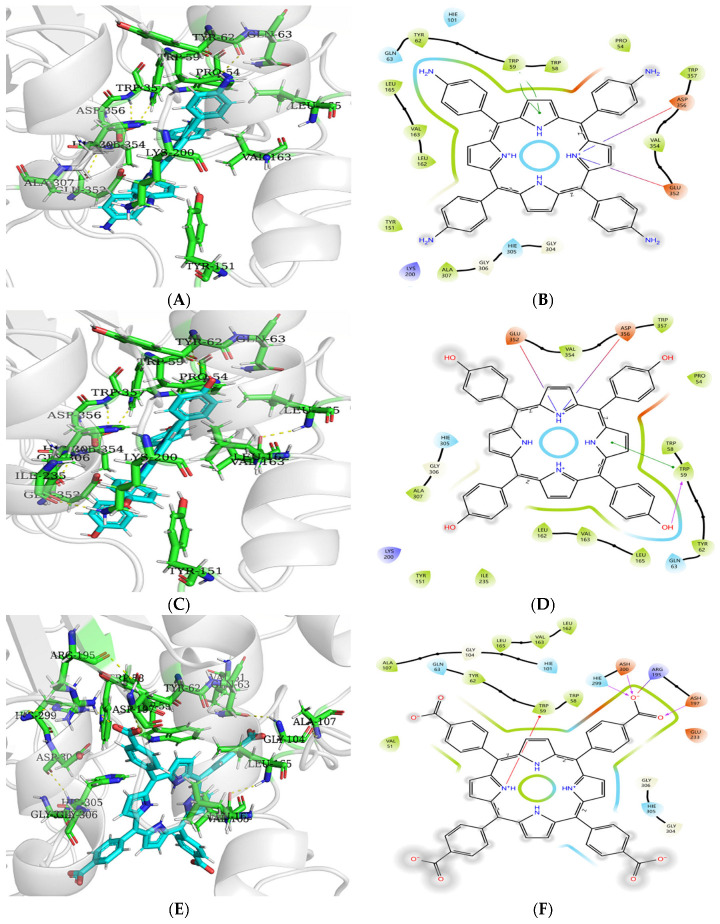
(**A**) Optimal pattern of interaction between TAPP and α-amylase; (**B**) 2D diagram; (**C**) optimal pattern of THPP interaction with α-amylase; (**D**) 2D diagram; (**E**) optimal pattern of TCPP interaction with α-amylase; (**F**) 2D diagram.

Figure 23 reveals that hydrophobic residues LEU162, VAL163, LEU165, TYR62, TRP59, and TRP58 of α-amylase envelop TAPP, THPP, and TCPP to form hydrophobic interactions. TAPP engages in π–π stacking with the aromatic residue TRP59, forms two salt bridges with ASP356 and GLU352, respectively, and establishes hydrophobic contacts with PRO54, TRP357, VAL354, ALA307, and TYR151. THPP donates a hydrogen bond from its phenolic hydroxyl group to TRP59, participates in π–π stacking with TRP59, forms salt bridges with ASP356 and GLU352, and develops hydrophobic interactions with PRO54, TRP357, VAL354, ALA307, TYR151, and ILE235. TCPP forms (i) a hydrogen bond between its carboxyl carbonyl oxygen and ASH197, (ii) hydrogen bonds between its carboxyl hydroxyl oxygen and both HIE299 and ASH300, and (iii) a salt bridge with ARG195. Although the carbonyl oxygen, hydroxyl oxygen, ASH197, and ASH300 carry partial negative charges that would theoretically cause electrostatic repulsion, the adjacent positively charged ARG195 likely neutralizes partial charges, reducing repulsive forces and permitting hydrogen bond formation. TCPP also exhibits a cation–π interaction with TRP59, attributable to electron-withdrawing carboxyl groups reducing the electron density of the porphyrin macrocycle. Conversely, electron-donating amino and hydroxyl groups in TAPP and THPP increase macrocycle electron density, enhancing π–π stacking stability with TRP59. Additionally, TCPP forms hydrophobic interactions with ALA107 and VAL51.

As shown in Table 11, more negative binding energy values indicate greater affinity. The molecular docking results of α-amylase with TAPP, THPP, and TCPP were −5.19, −6.82, and −2.90 kcal/mol, respectively, suggesting binding stability in the order of THPP > TAPP > TCPP. However, this differs from the previously observed inhibition ability order (TAPP > THPP > TCPP). The discrepancy may be attributed to differences between actual experimental conditions and theoretical docking calculations.

#### 3.9.2. Molecular Docking Analysis of Porphyrins with α-Glucosidase

**Figure 24:**
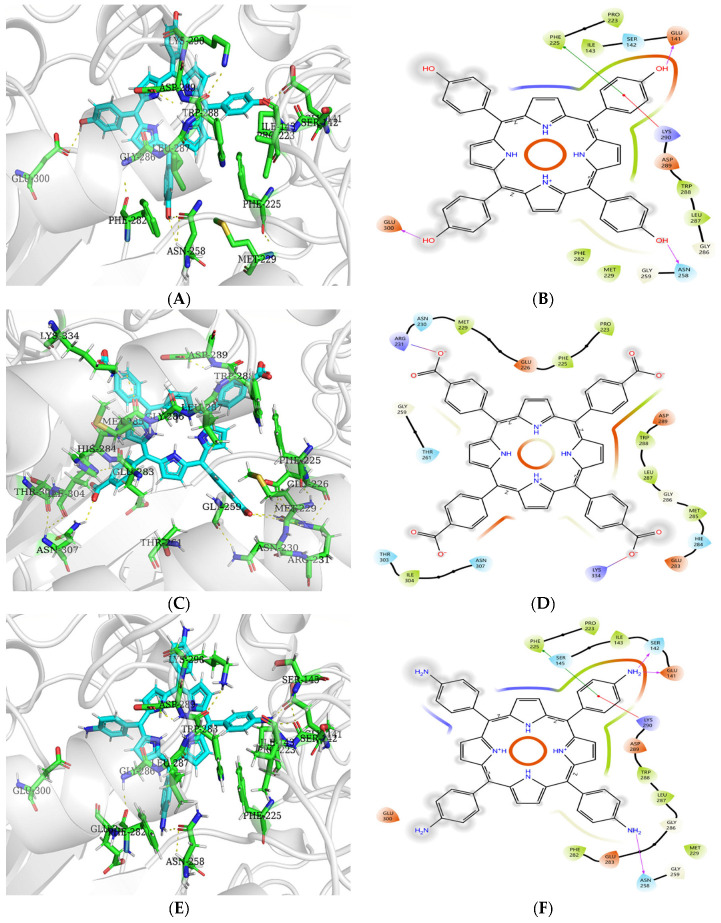
(**A**) Optimal pattern of THPP–α–glucosidase interaction; (**B**) 2D diagram; (**C**) optimal pattern of interaction between TCPP and α-glucosidase; (**D**) 2D diagram; (**E**) optimal pattern of TAPP interaction with α-glucosidase; (**F**) 2D diagram.

Figure 24 reveals that hydrophobic residues PRO223, PHE225, TRP288, LEU287, and MET229 enclose THPP, TCPP, and TAPP to form hydrophobic interactions. THPP establishes hydrogen bonds with GLU141, ASN258, and GLU300 via its phenolic hydroxyl group. Concurrently, it engages in π–π stacking with PHE225 and electrostatic interactions with the cationic side chain of LYS290. Adjacent hydrophobic residues ILE143 and PHE282 further contribute to hydrophobic stabilization. TCPP forms salt bridges between the hydroxyl oxygen of its carboxyl group and residues ARG231/LYS334. Hydrophobic contacts with MET285 and ILE304 enhance its binding. TAPP builds hydrogen bonds with SER142, GLU141, and ASN258 through its amino group. It exhibits π–π stacking with PHE225 and electrostatic attraction to LYS290’s cationic side chain. Proximity to hydrophobic residues ILE143 and PHE282 facilitates additional hydrophobic interactions.

As shown in Table 12, the binding free energies of α-GLU with THPP, TCPP, and TAPP were −6.39, −4.30, and −7.90 kcal/mol, respectively, indicating binding stability in the order of TAPP > THPP > TCPP, which contrasts with the experimentally determined inhibitory activity sequence THPP > TCPP > TAPP. This discrepancy, along with the absence of hydrogen bonding between α-glucosidase and TCPP, may be attributed to the theoretical vacuum conditions inherent in docking simulations. Such computational constraints deviate from actual experimental environments, consequently compromising the critical bridging role of water molecules. Furthermore, the observed inconsistency suggests that binding affinity derived from docking may not directly correlate with inhibitory potential.

Molecular docking is primarily based on static, thermodynamically dominated predictions of binding affinity, while experimentally measured inhibitory activities, such as IC_50_ and *Kᵢ* represent a more complex kinetic process, influenced by various factors including compound membrane permeability, solubility, and metabolic stability. Thus, the discrepancy between the binding affinity order from docking and the experimental inhibitory activity trend is a known phenomenon and not unique to this study.

Analysis of the structure–activity relationship of tetraphenylporphyrin derivatives bearing hydroxyl, amino, and carboxyl groups against three enzymes revealed that the hydroxyl group can form one and three hydrogen bonds with amino acid residues of α-amylase and α-glucosidase, respectively. The high absolute values of binding energy and strong inhibitory activity indicate that the hydroxyl group enhances the inhibition of both enzymes by forming hydrogen bonds with their amino acid residues. This may be attributed to the ability of the hydroxyl group to stabilize the electron cloud balance on the porphyrin macrocycle and increase its electron density. Meanwhile, the π–π stacking between the porphyrin macrocycle and amino acid residues improves the stability of the electron clouds in the α-amylase–THPP and α-glucosidase–THPP complexes, thereby strengthening the binding of THPP to the enzymes and enhancing its inhibitory activity. In contrast, the carboxyl group exhibited lower absolute values of both experimentally determined inhibitory activity and docking-simulated binding energy compared to the hydroxyl group. This may be due to the disruption of electron cloud balance by the carboxyl group, which reduces the electron density of the porphyrin macrocycle and destabilizes the π–π stacking. The amino group, however, can form three hydrogen bonds with α-glucosidase and a salt bridge with α-amylase, both of which play critical roles in stabilizing the enzyme–TAPP complex conformation. As an electron-donating group, the amino group increases the electron density on the porphyrin macrocycle and enhances the stability of π–π stacking, thereby contributing to the improved inhibitory activity.

### 3.10. Molecular Dynamics Simulation Analysis of Porphyrins and Enzymes

#### 3.10.1. Molecular Dynamics Simulation Analysis of Porphyrins and α-Amylase

**Figure 25:**
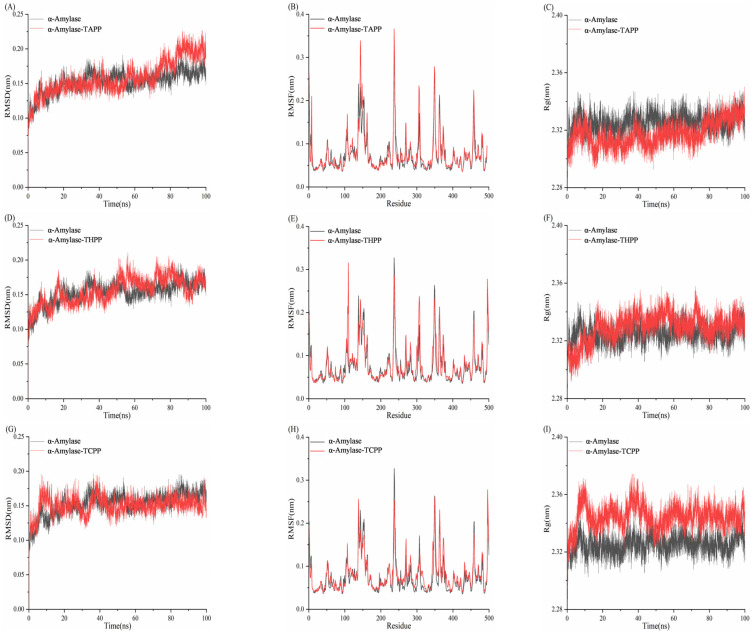
(**A**) RMSD diagram of TAPP interaction with α-amylase; (**B**) RMSF diagram; (**C**) Rg diagram; (**D**) RMSD plot of THPP interaction with α-amylase; (**E**) RMSF plot; (**F**) Rg plot; (**G**) RMSD plot of TCPP interaction with α-amylase; (**H**) RMSF plot; (**I**) Rg plot.

The Root Mean Square Deviation (RMSD) quantifies the positional divergence of a system’s conformation from its initial state during the simulation timeframe [47]. System stability is conventionally confirmed when RMSD fluctuations converge within the 0.10–0.30 nm range at simulation completion. Figure 25 demonstrates that all four systems maintained RMSD fluctuations between 0.10 and 0.25 nm, indicating attainment of equilibrium stability. The α-amylase–TAPP system exhibited lower RMSD fluctuations than free α-amylase within 0–70 ns but higher deviations during 70–100 ns, suggesting comparable overall stability. RMSD trajectories of the α-amylase–THPP system closely mirrored those of the unliganded enzyme, confirming similar dynamic stability. The α-amylase–TCPP complex displayed significantly reduced RMSD amplitudes relative to free α-amylase, indicative of enhanced structural stabilization. Residue-specific Root Mean Square Fluctuation (RMSF) analysis showed that most residues in ligand-bound complexes (TAPP/THPP/TCPP) exhibited higher flexibility than in free α-amylase. Select residues demonstrated reduced RMSF values, implying localized rigidification that contributes to structural integrity. Radius of Gyration (Rg) measurements further indicated that the α-amylase–TAPP complex achieved a lower Rg value than the apoenzyme, reflecting compact structural packing. Both α-amylase–THPP (+ΔRg) and α-amylase–TCPP (++ΔRg) complexes displayed increased Rg values relative to free α-amylase, signifying ligand-induced structural expansion and decreased compactness.

#### 3.10.2. Molecular Dynamics Simulation Analysis of Porphyrins and α-Glucosidase

**Figure 26:**
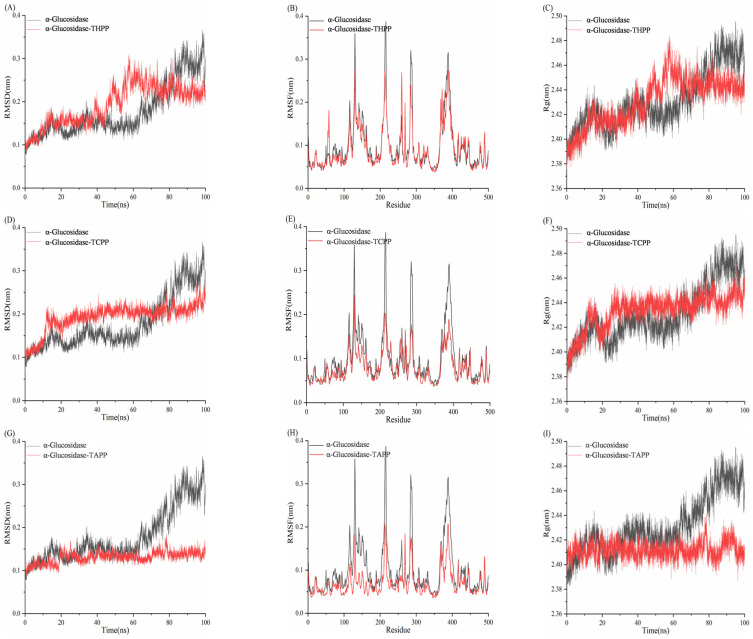
(**A**) RMSD plot of THPP interaction with α-glucosidase; (**B**) RMSF plot; (**C**) Rg plot; (**D**) RMSD plot of TCPP interaction with α-glucosidase; (**E**) RMSF plot; (**F**) Rg plot; (**G**) RMSD plot of TAPP interaction with α-glucosidase; (**H**) RMSF plot; (**I**) Rg plot.

In Figure 26, the RMSD values of α-glucosidase fluctuated between 0.10 and 0.18 nm within the first 65 ns. From 65 to 88 ns, the RMSD increased to 0.31 nm. During the 88–100 ns period, the RMSD initially decreased, then rose, and finally decreased to approximately 0.28 nm. The RMSD values of the α-glucosidase–THPP system ranged from 0.10 to 0.30 nm, indicating that the system reached equilibrium and exhibited greater stability compared to α-glucosidase alone. In the α-glucosidase–TCPP system, the RMSD increased to 0.20 nm within the first 12 ns and remained stable around 0.20 nm thereafter, demonstrating system equilibrium and higher stability than α-glucosidase. The RMSD of the α-GLU–TAPP system showed minimal fluctuation (0.10–0.15 nm), indicating significantly enhanced protein backbone stability upon complex formation with TAPP. For the α-GLU–THPP, α-GLU–TCPP, and α-GLU–TAPP complexes, the RMSF values of nearly all residues were lower than those of α-glucosidase alone. This demonstrates that all three complexes possess reduced flexibility and superior stability compared to the unbound enzyme. The Rg values of the three complexes stabilized at different time intervals: the α-glucosidase–THPP complex after 65 ns, the α-glucosidase–TCPP complex after 25 ns, and the α-glucosidase–TAPP complex throughout the entire 0–100 ns simulation. Notably, all three complexes exhibited lower Rg values than α-glucosidase, indicating more compact structural conformations.

### 3.11. Cytotoxicity Analysis of Porphyrins

**Figure 27:**
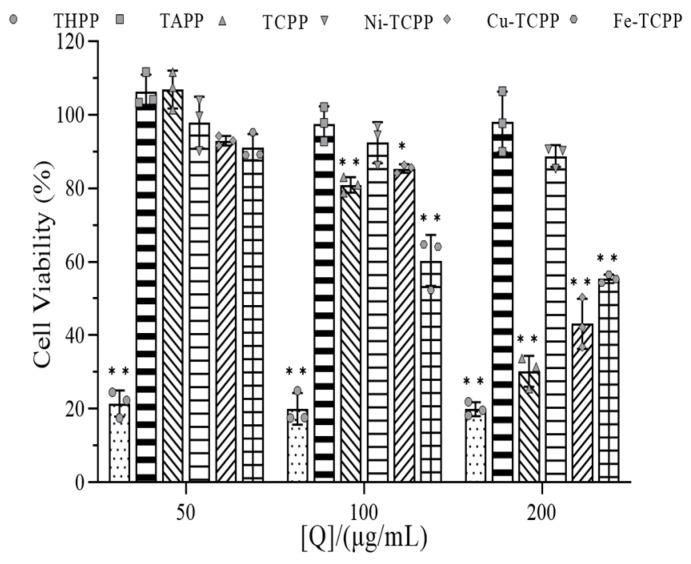
Effects of porphyrins on survival of HepG2 cells (* *p* < 0.05, ** *p* < 0.01) compared to blank controls.

Figure 27 shows the relationship between the concentration of six porphyrin derivatives and the survival rate of human hepatocellular carcinoma HepG2 cells. As the concentration of TAPP increased (50–200 μg/mL), the cell viability consistently exceeded 97%, indicating that TAPP exhibited no significant cytotoxicity toward HepG2 cells within the tested concentration range. Similarly, Ni–TCPP showed negligible toxicity to HepG2 cells at concentrations ranging from 50 to 200 μg/mL, with cell viability remaining above 88%. Both Cu–TCPP and TCPP demonstrated minimal toxicity at low concentrations (50–100 μg/mL), with cell viability rates above 80%. In contrast, Fe–TCPP caused no significant harm to HepG2 cells only at the very low concentration of 50 μg/mL.

When the concentration of Cu–TCPP and TCPP increased to 200 μg/mL, the survival rate of HepG2 cells decreased sharply to (43.13 ± 5.53%) and (30.26 ± 3.45%), respectively. For Fe–TCPP, at a concentration of 100 μg/mL, the cell viability was only (60.40 ± 5.69%). As the concentration further increased to 200 μg/mL, the viability declined to (55.41 ± 0.93%), a slower rate of decrease compared to Cu–TCPP and TCPP, indicating relatively lower cytotoxic activity.

THPP exhibited certain cytotoxic effects on HepG2 cells across the entire concentration range (50–200 μg/mL), demonstrating significant inhibitory and killing activity against liver cancer cells. This observed cytotoxicity may be attributed to two factors: firstly, the inherent toxicity of THPP within the 50–200 μg/mL concentration range, as well as that of high concentrations of Cu–TCPP, TCPP, and Fe–TCPP; secondly, the specific affinity of porphyrin compounds for cancer cells may enable selective accumulation within tumor cells, allowing them to act as antitumor photosensitizers in photodynamic therapy (PDT) to effectively kill cancer cells.

### 3.12. Pharmacokinetic Analysis Results

As shown in Table 13, human intestinal absorption (HIA) is an important parameter for evaluating the absorption characteristics of oral drug candidates. A positive absorption value indicates that the compound can be absorbed or assimilated through the intestine [48]. All six compounds exhibited HIA+, suggesting good potential for oral absorption. Moreover, blood–brain barrier penetration was positive (BBB+), indicating that these compounds can cross the blood–brain barrier during metabolism without causing harmful effects on the brain [49]. With the exception of TCPP, which showed a negative value, all other compounds were BBB+ and are thus not harmful to the brain. This study also included an assessment of acute oral toxicity as a predictive toxicological indicator. The results demonstrated that all six compounds belong to toxicity class III, indicating low acute oral toxicity.

## 4. Conclusions

α-Amylase and α-glucosidase are key digestive enzymes involved in blood glucose regulation. Inhibiting these enzymes can contribute to the prevention and management of diabetes. However, clinically used enzyme inhibitors often exhibit various adverse effects, making the search for alternative drugs to avoid or reduce these side effects a major research focus in the field of diabetes. Based on this context, this study investigates the inhibitory effects on these two enzymes, with the main conclusions summarized as follows.

Firstly, for α-amylase, the order of inhibitory effectiveness was TAPP > THPP > TCPP > Fe–TCPP > Ni–TCPP > Cu–TCPP. For α-glucosidase, the order was THPP > Ni–TCPP > Fe–TCPP > TCPP > Cu–TCPP > TAPP. Among these, TAPP and THPP showed stronger inhibition against α-amylase than acarbose. However, for α-glucosidase inhibition, all six compounds were less effective than acarbose, with THPP and Ni–TCPP being the closest to acarbose and still demonstrating strong inhibitory effects.

Research indicates that except for Ni–TCPP acting as a non-competitive inhibitor of α-amylase and THPP as a non-competitive inhibitor of α-glucosidase, the other compounds exhibit mixed-type inhibition toward both enzymes. Fluorescence quenching experiments further reveal that the binding between the compounds and the enzymes is primarily static quenching, resulting in the formation of ground-state complexes, with most compounds binding at a single site. Structure–activity relationship analysis demonstrates that different functional groups and metal centers significantly influence inhibitory activity and selectivity. For instance, TAPP and THPP, containing electron-donating groups (-NH_2_ and -OH), enhance π–π stacking interactions, leading to stronger inhibition of α-amylase. In contrast, the carboxyl groups in TCPP facilitate additional hydrogen bonding and salt bridge formation, though their inhibitory effects are relatively weaker. The introduction of metal ions generally reduces inhibitory activity, with the strength of inhibition correlated with the inherent characteristics of the ions. Furthermore, synchronous fluorescence and three-dimensional fluorescence spectroscopy show that compound binding alters the enzyme’s microenvironment and secondary structure, while infrared spectroscopy further confirms significant changes in enzyme conformation. Molecular docking and dynamics simulations not only validate the binding sites and the types of main interaction forces but also explain the differences in inhibitory effects among the compounds from the perspectives of energy and conformational stability. This study analyzed the specific inhibition mechanisms of porphyrin compounds on α-amylase and α-glucosidase and explored the binding mechanisms between porphyrin compounds and the enzymes, providing theoretical insights for the development of new antidiabetic drugs. However, certain limitations were identified during the research. For instance, drug development must consider not only in vitro inhibitory effects but also in vivo release and metabolism, necessitating further experimental investigations. Due to discrepancies between the molecular docking results and the inhibition order observed in earlier experiments, future studies could employ more precise molecular mechanics, such as Poisson–Boltzmann surface area (MM/PBSA) or molecular mechanics generalized Born surface area (MM/GBSA) calculations, on the initially docked complex structures to obtain more reliable binding free energy estimates, thereby improving the accuracy and consistency of binding affinity predictions. Additionally, time-resolved fluorescence spectroscopy could be applied to measure excited-state lifetimes and accurately distinguish between static and dynamic quenching. Furthermore, circular dichroism spectroscopy could be used in combination with the infrared spectroscopy mentioned in the text to comprehensively assess the proportions of protein secondary structures, enhancing the reliability of experimental data.

## Figures and Tables

**Table 1:** Inhibition kinetic constants of porphyrin compounds against α-amylase.

System	C (μg/mL)	*K_m_* (μg/mL)	*V*_max_ (ΔOD/min)	*K_i_* (μg/mL)	*K_is_* (μg/mL)
α-AMY–TAPP	0	3.9047 ± 0.1139	0.1826 ± 0.0053	8.7057 ± 0.0325	21.2847 ± 0.0677
	2	4.3893 ± 0.0776	0.1670 ± 0.0030		
	4	4.8719 ± 0.1211	0.1562 ± 0.0039		
	6	5.1449 ± 0.1158	0.1425 ± 0.0032		
	8	5.4460 ± 0.1386	0.1328 ± 0.0034		
	10	5.7081 ± 0.1284	0.1243 ± 0.0028		
α-AMY–THPP	0	3.6644 ± 0.0843	0.1390 ± 0.0032	9.2445 ± 0.0411	23.1012 ± 0.1027
	20	7.8640 ± 0.2930	0.0740 ± 0.0028		
	30	8.0378 ± 0.1371	0.0580 ± 0.0010		
	40	8.3899 ± 0.2135	0.0491 ± 0.0012		
	50	8.6487 ± 0.2634	0.0426 ± 0.0013		
	60	8.8467 ± 0.2802	0.0376 ± 0.0012		
α-AMY–TCPP	0	2.5848 ± 0.0724	0.1244 ± 0.0035	19.9216 ± 0.0524	132.4382 ± 0.1403
	25	4.9029 ± 0.1157	0.1046 ± 0.0025		
	50	6.3562 ± 0.1007	0.0872 ± 0.0014		
	100	8.8655 ± 0.2311	0.0709 ± 0.0018		
	150	10.3380 ± 0.3732	0.0583 ± 0.0021		
	200	11.3676 ± 0.1523	0.0496 ± 0.0007		
α-AMY–Fe–TCPP	0	2.4394 ± 0.0636	0.1387 ± 0.0036	69.5865 ± 0.1284	117.2152 ± 0.1432
	25	2.7329 ± 0.0882	0.1143 ± 0.0037		
	50	2.9384 ± 0.0447	0.0972 ± 0.0015		
	100	3.2079 ± 0.1016	0.0748 ± 0.0024		
	150	3.3360 ± 0.0508	0.0601 ± 0.0009		
	200	3.4920 ± 0.0491	0.0512 ± 0.0007		
α-AMY–Ni–TCPP	0	3.6368 ± 0.0487	0.1831 ± 0.0025	157.9542 ± 0.1375	
	40	3.6229 ± 0.1082	0.1456 ± 0.0043		
	80	3.6134 ± 0.1167	0.1238 ± 0.0040		
	120	3.6072 ± 0.0593	0.1032 ± 0.0017		
	160	3.6027 ± 0.1141	0.0901 ± 0.0029		
	200	3.5987 ± 0.0938	0.0799 ± 0.0021		
α-AMY–Cu–TCPP	0	2.5129 ± 0.0827	0.1155 ± 0.0038	135.8043 ± 0.1608	420.9420 ± 0.2189
	25	2.8021 ± 0.0410	0.1101 ± 0.0016		
	50	3.1685 ± 0.0729	0.1050 ± 0.0024		
	100	3.5949 ± 0.0569	0.0923 ± 0.0015		
	150	3.9016 ± 0.1355	0.0860 ± 0.0031		
	200	4.2470 ± 0.0673	0.0785 ± 0.0012		

**Table 2:** Inhibition kinetics of porphyrins on α-glucosidase.

System	C (μg/mL)	*K_m_* (μg/mL)	*V*_max_ (ΔOD/min)	*K_i_* (μg/mL)	*K_is_* (μg/mL)
α-Glucosidase–THPP	0	6.7070 ± 0.1543	0.2310 ± 0.0053	1.6508 ± 0.0135	
	1	7.1219 ± 0.0954	0.1528 ± 0.0020		
	2.5	7.3949 ± 0.2523	0.1013 ± 0.0035		
	5	7.5880 ± 0.2213	0.0649 ± 0.0019		
	7.5	7.6790 ± 0.1357	0.0489 ± 0.0009		
	10	7.7319 ± 0.1921	0.0377 ± 0.0009		
α-Glucosidase–Ni–TCPP	0	2.0514 ± 0.0462	0.1649 ± 0.0037	3.8703 ± 0.0201	9.2257 ± 0.0312
	5	3.0491 ± 0.0776	0.1036 ± 0.0026		
	7.5	3.3243 ± 0.0748	0.0909 ± 0.0020		
	10	3.5279 ± 0.0812	0.0791 ± 0.0018		
	15	3.8090 ± 0.1419	0.0628 ± 0.0023		
	25	4.1248 ± 0.0704	0.0444 ± 0.0008		
α-Glucosidase–Fe–TCPP	0	1.4377 ± 0.0366	0.1076 ± 0.0027	9.8917 ± 0.0514	40.4085 ± 0.1084
	16	2.6958 ± 0.0821	0.0771 ± 0.0023		
	32	3.3979 ± 0.1076	0.0601 ± 0.0019		
	48	3.9231 ± 0.1098	0.0502 ± 0.0014		
	64	4.1566 ± 0.0981	0.0416 ± 0.0010		
	80	4.3847 ± 0.0694	0.0361 ± 0.0006		
α-Glucosidase–TCPP	0	1.8019 ± 0.0470	0.1499 ± 0.0039	9.5746 ± 0.0611	52.5334 ± 0.1408
	5	2.5045 ± 0.0904	0.1369 ± 0.0049		
	25	4.4087 ± 0.0591	0.1016 ± 0.0014		
	50	5.5731 ± 0.1452	0.0745 ± 0.0019		
	100	7.1021 ± 0.2293	0.0516 ± 0.0017		
	150	7.7894 ± 0.1186	0.0389 ± 0.0006		
α-Glucosidase–Cu–TCPP	0	2.7842 ± 0.0882	0.1689 ± 0.0053	13.7332 ± 0.0823	38.9429 ± 0.1021
	5	3.3657 ± 0.0431	0.1496 ± 0.0019		
	10	3.8284 ± 0.1475	0.1344 ± 0.0052		
	25	4.7824 ± 0.1009	0.1028 ± 0.0022		
	50	5.4948 ± 0.1841	0.0718 ± 0.0024		
	100	6.4626 ± 0.1103	0.0473 ± 0.0008		
α-Glucosidase–TAPP	0	2.0896 ± 0.0305	0.0969 ± 0.0014	9.4635 ± 0.0572	55.1227 ± 0.1247
	12.5	3.9347 ± 0.1025	0.0791 ± 0.0021		
	15	4.2527 ± 0.0701	0.0767 ± 0.0013		
	17.5	4.5048 ± 0.0928	0.0736 ± 0.0015		
	20	4.7611 ± 0.0754	0.0711 ± 0.0011		
	22.5	5.0007 ± 0.0856	0.0688 ± 0.0012		

**Table 3:** Fluorescence quenching constants, binding constants, and number of binding sites for porphyrin interactions with α-amylase.

System	T	*K_sv_* (×10^4^ L/mol)	*K_q_* (×10^12^ L/mol/s)	*n*	*K_a_* (×10^5^ L/mol)
α-Amylase–TAPP	298	4.07 ± 0.05	4.07 ± 0.05	1.48	52.90 ± 0.07
	304	3.37 ± 0.02	3.37 ± 0.02	1.40	21.79 ± 0.07
	310	3.07 ± 0.09	3.07 ± 0.09	1.31	7.46 ± 0.02
α-Amylase–THPP	298	8.90 ± 0.06	8.90 ± 0.06	1.19	5.65 ± 0.06
	304	8.20 ± 0.02	8.20 ± 0.02	1.16	3.65 ± 0.03
	310	7.64 ± 0.07	7.64 ± 0.07	1.12	2.46 ± 0.01
α-Amylase–TCPP	298	4.04 ± 0.01	4.04 ± 0.01	1.36	12.54 ± 0.05
	304	3.55 ± 0.08	3.55 ± 0.08	1.28	5.01 ± 0.03
	310	3.01 ± 0.07	3.01 ± 0.07	1.20	2.18 ± 0.02
α-Amylase–Fe–TCPP	298	4.32 ± 0.06	4.32 ± 0.06	1.65	944.30 ± 0.18
	304	3.36 ± 0.06	3.36 ± 0.06	1.38	40.38 ± 0.09
	310	3.29 ± 0.08	3.29 ± 0.08	1.05	2.13 ± 0.05
α-Amylase–Ni–TCPP	298	9.78 ± 0.03	9.78 ± 0.03	1.44	99.31 ± 0.12
	304	8.47 ± 0.07	8.47 ± 0.07	1.19	5.75 ± 0.04
	310	7.11 ± 0.05	7.11 ± 0.05	0.99	0.61 ± 0.02
α-Amylase–Cu–TCPP	298	4.37 ± 0.07	4.37 ± 0.07	0.93	0.20 ± 0.01
	304	4.33 ± 0.03	4.33 ± 0.03	0.84	0.10 ± 0.01
	310	4.32 ± 0.02	4.32 ± 0.02	0.78	0.06 ± 0.01

**Table 4:** Fluorescence quenching constants, binding constants, and number of binding sites for porphyrin interactions with α-glucosidase.

System	T	*K_sv_* (×10^4^ L/mol)	*K_q_* (×10^12^ L/mol/s)	*n*	*K_a_* (×10^5^ L/mol)
α-Glucosidase–THPP	298	7.40 ± 0.06	7.40 ± 0.06	1.29	15.49 ± 0.08
	304	6.97 ± 0.06	6.97 ± 0.06	1.23	8.01 ± 0.06
	310	6.69 ± 0.04	6.69 ± 0.04	1.16	3.39 ± 0.03
α-Glucosidase–Ni–TCPP	298	17.73 ± 0.15	17.73 ± 0.15	1.03	2.34 ± 0.04
	304	14.23 ± 0.09	14.23 ± 0.09	1.04	2.09 ± 0.02
	310	12.31 ± 0.08	12.31 ± 0.08	1.03	1.83 ± 0.01
α-Glucosidase–Fe–TCPP	298	14.52 ± 0.07	14.52 ± 0.07	1.01	1.65 ± 0.03
	304	13.18 ± 0.05	13.18 ± 0.05	1.00	1.26 ± 0.03
	310	12.20 ± 0.07	12.20 ± 0.07	0.98	1.02 ± 0.02
α-Glucosidase–TCPP	298	7.45 ± 0.04	7.45 ± 0.04	1.30	13.73 ± 0.06
	304	4.90 ± 0.02	4.90 ± 0.02	1.19	3.17 ± 0.03
	310	4.22 ± 0.09	4.22 ± 0.09	1.07	0.97 ± 0.02
α-Glucosidase–Cu–TCPP	298	7.35 ± 0.09	7.35 ± 0.09	1.44	55.21 ± 0.11
	304	6.74 ± 0.03	6.74 ± 0.03	1.25	7.38 ± 0.04
	310	5.05 ± 0.02	5.05 ± 0.02	1.03	0.69 ± 0.01
α-Glucosidase–TAPP	298	6.25 ± 0.04	6.25 ± 0.04	1.14	2.78 ± 0.01
	304	5.71 ± 0.01	5.71 ± 0.01	1.09	1.42 ± 0.03
	310	5.66 ± 0.02	5.66 ± 0.02	1.01	0.65 ± 0.02

**Table 5:** Thermodynamic parameters of porphyrin interactions with α-amylase.

System	T	ΔH^0^ (kJ/mol)	ΔG^0^ (kJ/mol)	ΔS^0^ (J/mol/k)	R^2^
α-AMY– TAPP	298	−125.25 ± 0.12	−38.36 ± 0.11	−291.29 ± 0.12	0.99
	304		−36.89 ± 0.12		
	310		−34.85 ± 0.10		
α-AMY–THPP	298	−53.25 ± 0.05	−32.82 ± 0.08	−68.61 ± 0.06	0.99
	304		−32.37 ± 0.08		
	310		−31.99 ± 0.07		
α-AMY–TCPP	298	−112.04 ± 0.06	−34.79 ± 0.09	−259.31 ± 0.09	0.99
	304		−33.17 ± 0.07		
	310		−31.68 ± 0.07		
α-AMY–Fe–TCPP	298	−390.15 ± 0.17	−45.50 ± 0.07	−1156.68 ± 0.13	0.99
	304		−38.45 ± 0.06		
	310		−31.62 ± 0.08		
α-AMY–Ni–TCPP	298	−326.36 ± 0.18	−39.92 ± 0.08	−961.90 ± 0.08	0.99
	304		−33.52 ± 0.06		
	310		−28.39 ± 0.07		
α-AMY–Cu–TCPP	298	−79.68 ± 0.07	−24.55 ± 0.05	−185.16 ± 0.05	0.99
	304		−23.29 ± 0.03		
	310		−22.33 ± 0.05		

**Table 6:** Thermodynamic parameters of porphyrin interactions with α-glucosidase.

System	T	ΔH^0^ (kJ/mol)	ΔG^0^ (kJ/mol)	ΔS^0^ (J/mol/k)	R^2^
α-GLU–THPP	298	−97.17 ± 0.09	−35.31 ± 0.10	−207.25 ± 0.13	0.98
	304		−34.36 ± 0.09		
	310		−32.82 ± 0.06		
α-GLU–Ni–TCPP	298	−15.84 ± 0.05	−30.63 ± 0.13	49.67 ± 0.08	0.99
	304		−30.96 ± 0.06		
	310		−31.23 ± 0.07		
α-GLU–Fe–TCPP	298	−30.90 ± 0.06	−29.76 ± 0.05	−3.88 ± 0.01	0.99
	304		−29.68 ± 0.07		
	310		−29.72 ± 0.06		
α-GLU–TCPP	298	−169.47 ± 0.10	−35.01 ± 0.06	−451.52 ± 0.12	0.99
	304		−32.01 ± 0.07		
	310		−29.60 ± 0.05		
α-GLU–Cu–TCPP	298	−280.76 ± 0.13	−38.46 ± 0.08	−812.45 ± 0.17	0.99
	304		−34.15 ± 0.05		
	310		−28.70 ± 0.04		
α-GLU–TAPP	298	−93.02 ± 0.08	−31.06 ± 0.08	−207.76 ± 0.14	0.99
	304		−29.98 ± 0.05		
	310		−28.56 ± 0.05		

**Table 7:** 3D fluorescence spectroscopy data for α-amylase and α-amylase–porphyrin complexes.

System	Peak 1 Ex/Em (nm)	Fluorescence Intensity	Peak 2 Ex/Em (nm)	Fluorescence Intensity
α-Amylase (A)	280.0/356.0	364.8	236.0/352.0	8.688
α-Amylase–TAPP(A′)	280.0/348.0	317.2	236.0/352.0	7.013
α-Amylase(B/C)	280.0/352.0	608.4	236.0/352.0	85.34
α-Amylase–THPP(B′)	280.0/352.0	297.5	236.0/352.0	32.69
α-Amylase–TCPP(C′)	280.0/352.0	461.6	236.0/352.0	58.06
α-Amylase (D)	280.0/356.0	373.4	236.0/356.0	48.99
α-Amylase–Fe–TCPP(D′)	280.0/352.0	270.8	236.0/356.0	28.26
α-Amylase (E/F)	280.0/356.0	376.4	236.0/356.0	23.03
α-Amylase–Ni–TCPP(E′)	280.0/356.0	225.2	236.0/360.0	9.012
α-Amylase–Cu–TCPP(F′)	280.0/352.0	313.1	236.0/356.0	16.48

**Table 8:** Three-dimensional fluorescence spectroscopic data for α-glucosidase and α-glucosidase–porphyrin complexes.

System	Peak 1 Ex/Em (nm)	Fluorescence Intensity	Peak 2 Ex/Em (nm)	Fluorescence Intensity
α-Glucosidase(A/C/D)	280.0/348.0	207.4	236.0/356.0	33.69
α-Glucosidase–THPP(A′)	280.0/348.0	122.3	236.0/352.0	13.28
α-Glucosidase–Fe–TCPP(C′)	280.0/344.0	168.0	236.0/352.0	16.79
α-Glucosidase–TCPP(D′)	280.0/348.0	160.0	236.0/352.0	22.17
α-Glucosidase (B/E)	280.0/348.0	205.7	236.0/356.0	22.99
α-Glucosidase–Ni–TCPP(B′)	280.0/352.0	101.6	236.0/352.0	6.157
α-Glucosidase–Cu–TCPP(E′)	280.0/348.0	139.0	236.0/356.0	6.458
α-glucosidase (F)	280.0/352.0	216.1	236.0/352.0	8.943
α-Glucosidase–TAPP(F′)	280.0/352.0	159.0	236.0/348.0	0.576

**Table 9:** Secondary structural analysis of α-AMY and α-AMY–porphyrin complexes.

System	α-Helix (%)	β-Sheet (%)	β-Turn (%)	Random Coil (%)	β-Antiparallel (%)
α-AMY	24.58 ± 0.03	23.64 ± 0.07	27.34 ± 0.06	14.10 ± 0.04	10.34 ± 0.02
α-AMY–TAPP	22.45 ± 0.07	14.62 ± 0.03	34.00 ± 0.09	9.94 ± 0.02	18.98 ± 0.04
α-AMY–THPP	22.24 ± 0.06	12.92 ± 0.05	33.76 ± 0.08	17.71 ± 0.07	13.37 ± 0.03
α-AMY–TCPP	10.24 ± 0.02	18.51 ± 0.05	39.66 ± 0.09	19.84 ± 0.06	11.75 ± 0.03
α-AMY–Fe–TCPP	21.77 ± 0.07	12.86 ± 0.04	34.02 ± 0.08	22.59 ± 0.03	8.76 ± 0.02
α-AMY–Ni–TCPP	20.76 ± 0.06	11.20 ± 0.02	36.33 ± 0.13	21.40 ± 0.05	10.30 ± 0.03
α-AMY–Cu–TCPP	20.76 ± 0.05	10.94 ± 0.01	35.19 ± 0.06	20.94 ± 0.06	12.17 ± 0.02

**Table 10:** Secondary structural analysis of α-GLU and α-GLU–porphyrin complexes.

System	α-Helix (%)	β-Sheet (%)	β-Turn (%)	Random Coil (%)	β-Antiparallel (%)
α-GLU	24.74 ± 0.03	14.72 ± 0.04	32.91 ± 0.09	19.42 ± 0.07	8.21 ± 0.03
α-GLU–THPP	21.19 ± 0.05	12.79 ± 0.03	35.20 ± 0.11	23.74 ± 0.06	7.07 ± 0.01
α-GLU–Ni–TCPP	19.75 ± 0.07	13.68 ± 0.04	37.94 ± 0.10	26.76 ± 0.07	1.88 ± 0.01
α-GLU–Fe–TCPP	22.48 ± 0.05	15.77 ± 0.04	36.97 ± 0.08	16.75 ± 0.03	8.02 ± 0.02
α-GLU–TCPP	22.71 ± 0.05	12.03 ± 0.02	33.54 ± 0.07	22.15 ± 0.04	9.57 ± 0.02
α-GLU–Cu–TCPP	23.65 ± 0.03	10.93 ± 0.05	37.78 ± 0.09	25.05 ± 0.07	2.59 ± 0.01
α-GLU–TAPP	22.82 ± 0.08	12.99 ± 0.13	38.30 ± 0.08	23.93 ± 0.05	1.96 ± 0.01

**Table 11:** Molecular docking results of α-amylase with porphyrins.

System	XP GScore (kcal/mol)	Key Residues	Hydrogen Bonds
α-amylase–TAPP	−5.19	LEU162, VAL163, LEU165, TYR62, TRP59, TRP58, PRO54, TRP357, ASP356, VAL354, GLU352, ALA307, TYR151	
α-amylase–THPP	−6.82	LEU162, VAL163, LEU165, TYR62, TRP59, TRP58, PRO54, TRP357, ASP356, VAL354, GLU352, ALA307, TYR151, ILE235	TRP59
α-amylase–TCPP	−2.90	LEU162, VAL163, LEU165, TYR62, TRP59, TRP58, ALA107, HIE299, ASH300, ARG195, ASH197, VAL51	HIE299, ASH300, ASH197

**Table 12:** Molecular docking results of α-glucosidase to porphyrins.

System	XP GScore (kcal/mol)	Key Residues	Hydrogen Bonds
α-Glucosidas–THPP	−6.39	PRO223, PHE225, ILE143, GLU141, LYS290, TRP288, LEU287, ASN258, MET229, PHE282, GLU300	GLU141, ASN258, GLU300
α-Glucosidase–TCPP	−4.30	PRO223, PHE225, MET229, ARG231, TRP288, LEU287, MET285, LYS334, ILE304	
α-Glucosidase–TAPP	−7.90	PRO223, PHE225, ILE143, SER142, GLU141, LYS290, TRP288, LEU287, PHE282, ASN258, MET229	SER142, GLU141, ASN258

**Table 13:** ADMET analysis of compounds.

Compound	BBB	HIA	Oral Toxicity
THPP	0.5000+	0.9951+	(III)0.5450
TAPP	0.7799+	0.9955+	(III)0.6175
TCPP	0.5357−	0.9739+	(III)0.5439
Fe–TCPP	0.5795+	0.8931+	(III)0.5356
Ni–TCPP	0.5335+	0.7102+	(III)0.5504
Cu–TCPP	0.5674+	0.7426+	(III)0.5504

## Data Availability

All data included in this study are available upon request by contacting the corresponding author.

## Abbreviations

The following abbreviations are used in this manuscript:

α-AMY	α-amylas
α-GLU	α-glucosidase
